# DNA Barcoding the Geometrid Fauna of Bavaria (Lepidoptera): Successes, Surprises, and Questions

**DOI:** 10.1371/journal.pone.0017134

**Published:** 2011-02-14

**Authors:** Axel Hausmann, Gerhard Haszprunar, Paul D. N. Hebert

**Affiliations:** 1 Entomology Department, Zoological Collection of the State of Bavaria, Munich, Germany; 2 Biodiversity Institute of Ontario, University of Guelph, Guelph, Canada; Biodiversity Insitute of Ontario - University of Guelph, Canada

## Abstract

**Background:**

The State of Bavaria is involved in a research program that will lead to the construction of a DNA barcode library for all animal species within its territorial boundaries. The present study provides a comprehensive DNA barcode library for the Geometridae, one of the most diverse of insect families.

**Methodology/Principal Findings:**

This study reports DNA barcodes for 400 Bavarian geometrid species, 98 per cent of the known fauna, and approximately one per cent of all Bavarian animal species. Although 98.5% of these species possess diagnostic barcode sequences in Bavaria, records from neighbouring countries suggest that species-level resolution may be compromised in up to 3.5% of cases. All taxa which apparently share barcodes are discussed in detail. One case of modest divergence (1.4%) revealed a species overlooked by the current taxonomic system: Eupithecia goossensiata Mabille, 1869 stat.n. is raised from synonymy with Eupithecia absinthiata (Clerck, 1759) to species rank. Deep intraspecific sequence divergences (>2%) were detected in 20 traditionally recognized species.

**Conclusions/Significance:**

The study emphasizes the effectiveness of DNA barcoding as a tool for monitoring biodiversity. Open access is provided to a data set that includes records for 1,395 geometrid specimens (331 species) from Bavaria, with 69 additional species from neighbouring regions. Taxa with deep intraspecific sequence divergences are undergoing more detailed analysis to ascertain if they represent cases of cryptic diversity.

## Introduction

Bavaria, the largest federal state of Germany, is situated in the very center of Europe. Despite this fact, it still lacks a comprehensive faunistic monograph for its more than 3,200 Lepidoptera species. The classic fauna of Osthelder [1] is seriously outdated, covers only the southern half of the territory and is greatly biased towards Macrolepidoptera. An updated faunal list can be inferred through the German faunal list [2], but such checklists are no substitute for faunistic assessments. The neighbouring federal state, Baden-Württemberg, has produced a splendid series of monographs on its Lepidoptera fauna [3] which is exemplary and comprehensive. However, despite more than 20 years of work by hundreds of volunteers, and despite substantial financial support, the Microlepidoptera were excluded, though they represent the majority of Lepidoptera species. Given the obvious limits of traditional methods for biodiversity assessment, and encouraged by the recent development of DNA barcoding as an alternative approach for both the identification of described species and the discovery of new ones [4]–[6], DNA barcoding was adopted as an alternative strategy for the rapid, cost-effective assessment of the Bavarian fauna.

The project ‘Barcoding Fauna Bavarica (BFB)’ was activated in 2009 supported by a 5-year grant from the Bavarian State Government [7]. Research activities involve close cooperation with the Biodiversity Institute of Ontario, which performs the sequence analyses under the framework of the International Barcode of Life Project (iBOL). BFB represents the first effort to create a DNA barcode library for all animal species in a whole country. The first project period seeks to achieve coverage for at least 10,000 species by the end of 2013 [7]. By October 2010, barcode records were available for 10,000 Bavarian specimens representing more than 4,000 species [8], of which about 2,100 are Lepidoptera. Coverage is at 11 per cent for the complete Bavarian animal fauna, and 66 per cent for Bavarian Lepidoptera.

Due to its high diversity of habitat types, including alpine habitats and lowland stream valleys, Bavaria hosts approximately 90 per cent of the continental species known from Germany [7], 70 per cent of the fauna of Central Europe and about one third of the European fauna. Therefore the BFB project has a great potential to impact European zoology.

Reflecting a long illustrious entomological tradition starting with Bavarian lepidopterists like Hübner, Esper, Schrank and Herrich-Schäffer, the macrolepidopteran fauna of Bavaria is generally thought to be completely known. The assembly of a DNA library for Bavarian geometrids provides an opportunity to test this conclusion because the sequence data provide an additional character set to verify or reject taxonomic concepts (species delimitations, possible synonymies etc.) in an integrated taxonomic approach.

This paper provides open access to almost all of our data on Bavarian Geometridae. Such data releases in the Barcode of Life Data System (BOLD) and GenBank represent an important contribution to the democratization of biodiversity information because each barcode record is accompanied by georeferenced data and images of its source specimen [9]–[11].

## Materials and Methods

### Sampling

DNA barcodes were obtained by sampling dry legs from specimens in the Bavarian State Collection of Zoology (ZSM) and some private collections, e.g. of Alfred Haslberger (Teisendorf) and Theo Grünewald (Landshut). Sampling was usually restricted to a few vouchers per species, trying to include material from all four major Bavarian fauna regions. As a consequence, potential sampling biases due to constrained geographical coverage are expected to play a negligible role [cf 12]. By early 2010, tissue samples from 1,818 Bavarian geometrids had been submitted for DNA barcoding. All specimens were identified by the senior author, and dissections were made in all difficult cases. Taxonomy and nomenclature (see Appendix S1, Appendix S2) is based on an internal, updated faunistic database (A. Segerer pers. comm.) that reflects taxonomic decisions by Scoble & Hausmann [13]. A website has been established for ‘Barcoding Fauna Bavarica’ [8] that continuously updates project progress, such as lists of species that lack barcode coverage.

At present, there are barcodes for 400 of the 407 Bavarian geometrid species in the Barcode of Life Data System (BOLD; see Appendix S1). The following three species are entirely missing: *Alsophila aceraria* (Denis & Schiffermüller, 1775), *Idaea contiguaria* (Hübner, 1799), *Scopula nemoraria* (Hübner, 1799). Four other species are under analysis: *Artiora evonymaria* (Denis & Schiffermüller, 1775), *Cabera leptographa* Wehrli, 1936, *Stegania cararia* (Hübner, 1790), *Perizoma lugdunaria* (Herrich-Schäffer, 1855).

### DNA Analysis

PCR amplification and DNA sequencing were performed at the Canadian Centre for DNA Barcoding following standard high-throughput protocols [14]–[15], that can be accessed under http://www.dnabarcoding.ca/pa/ge/research/protocols. PCR amplification with a single pair of primers consistently recovered a 658 bp region near the 5′ terminus of the mitochondrial cytochrome *c* oxidase I (COI) gene that included the standard 648 bp barcode region for the animal kingdom [4]. All barcoded voucher specimens are listed in Appendix S1. DNA extracts are currently stored at the Canadian Centre for DNA Barcoding, but aliquots will also be deposited in the DNA-Bank facility of the ZSM (see http://www.zsm.mwn.de/dnabank/). All new sequences were deposited in GenBank according to the data release policy of the International Barcode of Life Project, accession numbers are given in Appendix S3. Complete specimen data including images, voucher deposition, GenBank accession numbers, GPS coordinates, sequence and trace files can easily be accessed in the Barcode of Life Data System [9], [16] in three public projects (FBLGE, FBLGL, FBLGO). Access has been restricted until 2011 for a very few species that show deep intraspecific divergences to enable additional studies aimed at clarifying their status.

### Data Analysis

Sequence divergences for the barcode region were calculated using the Kimura 2 Parameter model, employing the analytical tools on BOLD. Genetic distances between species are reported as minimum pairwise distances, while intraspecific variation is reported as maximum pairwise distances.

## Results

A sequence record was obtained from 1,395 of 1,818 specimens (77%) submitted for analysis representing 331 species of which 1,321 (325 species) records were longer than 500 bp. A 658 bp record was obtained from 921 of these specimens (303 species). In the following analysis, additional data from 69 species are included (see Appendix S1), which are found in Bavaria, but which are currently only represented by specimens from other localities in Europe.

### Unambiguous Data and Interspecific Genetic Distances

The COI barcode sequences for at least 96.5 per cent of Bavarian geometrid species are distinct from those of any other closely related species recognized through classic entomological approaches. The remaining 12 species, which may share barcodes, are discussed in the next two sections. Twenty other species showed deep intraspecific splits (see below), but these never lead to misidentifications, because none of the sequences overlap with those in any other species.

The mean genetic distance between Bavarian geometrid species averages 13.3 per cent (SE  =  0.003; n = 385,756 comparisons in the analysis of full-length barcodes), while congeneric species average 10.0 per cent divergence (SE = 0.014; n = 18,603 comparisons in the analysis of full-length barcodes). Table 1 provides a list of the 15 species pairs with the lowest divergence values.

**Table 1:** A list of the 15 pairs of Bavarian geometrid species with a minimum pairwise distance (K2P) of 1.0 to 4.0%.

species 1	species 2	min p.d. %
See chapter ‘cases of barcode sharing’	See chapter ‘cases of barcode sharing’	<1%
*Thera variata* (Denis & Schiffermüller, 1775)	*Thera britannica* (Turner, 1925)	1.1
*Eupithecia absinthiata* (Clerck, 1759)	*Eupithecia goossensiata* Mabille, 1869	1.4
*Chloroclysta siterata* (Hufnagel, 1767)	*Chloroclysta miata* (Linnaeus, 1758) *	1.5
*Eupithecia semigraphata* Bruand, 1850	*Eupithecia impurata* (Hübner, 1813)	1.9
*Thera britannica* (Turner, 1925)	*Thera vetustata* (Denis & Schiffermüller, 1775)	2.2
*Eupithecia subumbrata* (Denis & Schiffermüller, 1775)	*Eupithecia orphnata* Petersen, 1909	2.3
*Thera variata* (Denis & Schiffermüller, 1775)	*Thera vetustata* (Denis & Schiffermüller, 1775)	2.4
*Ennomos quercinaria* (Hufnagel, 1767)	*Ennomos autumnaria* (Werneburg, 1859)	2.5
*Entephria nobiliaria* (Herrich-Schäffer, 1852)	*Entephria flavata* (Osthelder, 1929)	2.7
*Eupithecia linariata* (Denis & Schiffermüller, 1775)	*Eupithecia pulchellata* Stephens, 1831	2.7
*Epirrhoe alternata* (Müller, 1764)	*Epirrhoe rivata* (Hübner, 1813)	3.5
*Cyclophora punctaria* (Linnaeus, 1758)	*Cyclophora albipunctata* (Hufnagel, 1767)	3.5
*Cyclophora linearia* (Hübner, 1799)	*Cyclophora albipunctata* (Hufnagel, 1767)	3.5
*Idaea humiliata* (Hufnagel, 1767)	*Idaea dilutaria* (Hübner, 1799)	3.8
*Horisme tersata* (Denis & Schiffermüller, 1775)	*Horisme radicaria* (Harpe, 1855)	3.9
All other species	All other species	>4

All results are based on comparison of 658 bp amplicons. min p.d.  =  minimum pairwise distance. * The analysis for *Chloroclysta miata* was performed with extraterritorial data.

**Figure 1:**
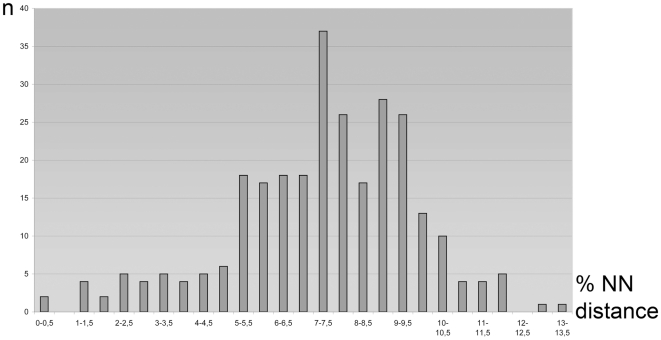
Nearest-Neighbour (NN) K2P distances for 283 Bavarian geometrid species. Histogram of species numbers (n) by 0.5 per cent-classes. This analysis is based on a comparison of 658 bp barcode records from the study area. Twenty additional species with intra-specific divergences greater than two per cent were excluded.

**Figure 2:**
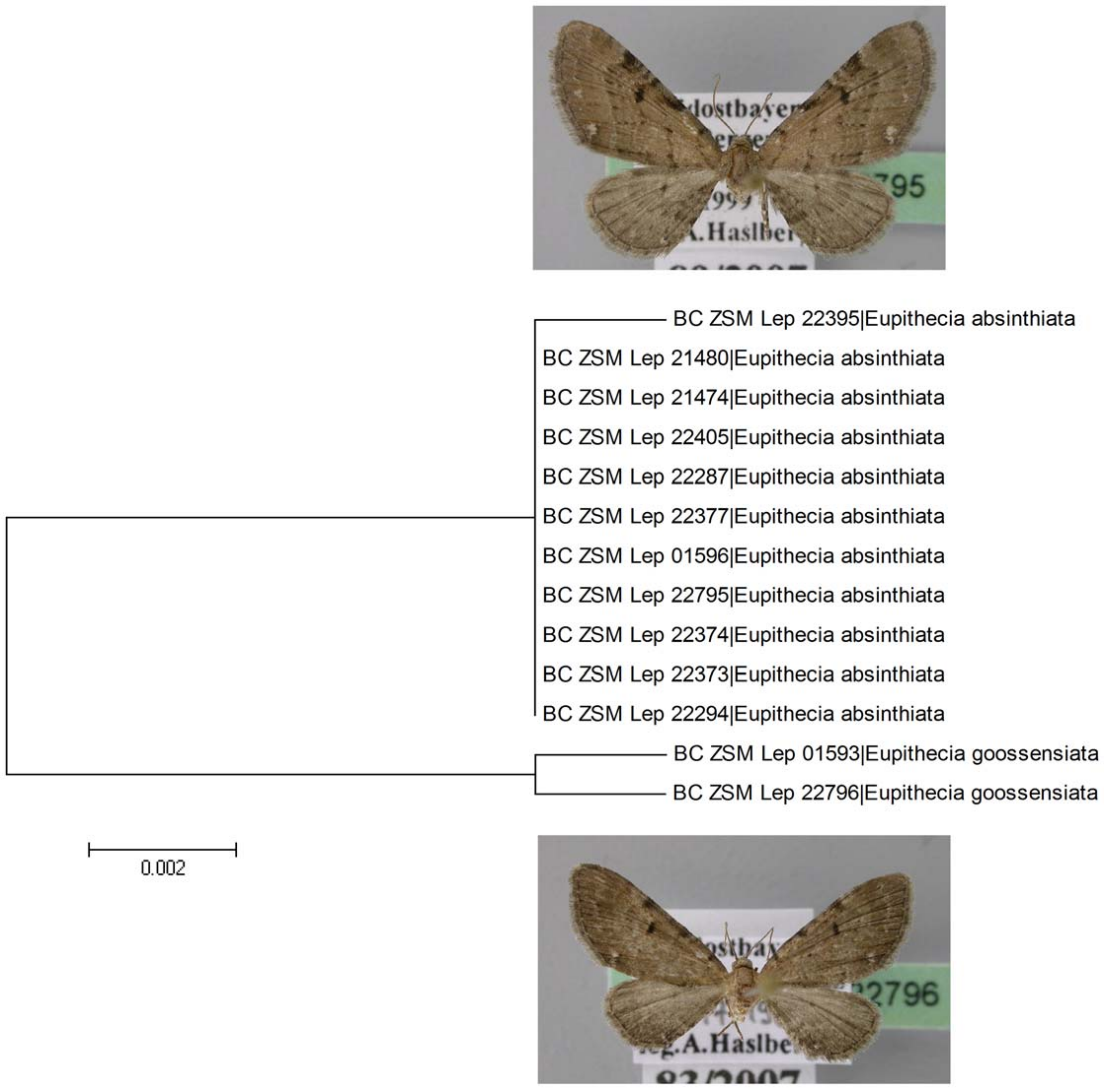
Neighbour joining tree (K2P) for Bavarian specimens of Eupithecia absinthiata and E. goossensiata. Branches with specimen ID-number from BOLD and species name. The consistent barcode divergence of 1.4% coupled with morphological and ecological differences confirms that these taxa are different species.

Only 25 (6.25%) of currently recognized Bavarian geometrid species showed less than 3% sequence divergence from their nearest neighbour. These cases involve the 17 species listed in Table 1 (10 species pairs with 3 species showing up twice) and in possibly four other species pairs in the genera *Chlorissa*, *Lycia*, *Perizoma*, and *Sciadia/Elophos* that are discussed in a later section. Just eight of these pairs diverge by less than 2%, four listed in Table 1 and four others discussed in detail below (cf. Figure 1). One of these pairs, *Eupithecia absinthiata* (Clerck, 1759) and *Eupithecia goossensiata* Mabille, 1869, show a constant genetic divergence of 1.4% in 15 specimens from different localities in Bavaria (Figure 2). The status of these two species was uncertain until the present study [3], [17]. The species do show slight differences in wing coloration and size, and ecological differences as the latter species is restricted to bogs and heather moorland while the former is found in a large variety of mesophilic open and semi-open habitats. However, because detailed examination of genitalic traits did not reveal any differential features, *Eupithecia goossensiata* was downgraded to synonymy in a recent monograph on European geometrids [17]. Because of the covariation between barcode divergence, habitat differences, and morphological differences, we reverse this conclusion, and recognize *E. goossensiata* as a valid species (*Eupithecia goossensiata* Mabille, 1869 stat.n.).

The status of another species pair, *Entephria nobiliaria* (Herrich-Schäffer, 1852) and *E. flavata* (Osthelder, 1929) has also been controversial. Both taxa were recognized in the Fauna of Europe database [18], a conclusion which gains confirmation from 2.7% barcode divergence between them (Table 1).

### Cases of Barcode Sharing or Low Divergence

Only one species in Bavaria was found to regularly and exactly share its barcode sequence with another morphologically distinct species. This case involved:

#### Chlorissa viridata (Linnaeus, 1758) – Chlorissa cloraria (Hübner, 1813)

Discrimination of this pair of sibling species is very challenging even by traditional methods and requires a multivariate analysis of characters in wing coloration and genitalia [19]. One dissected and phenotypically unambiguous southern Bavarian male of *C. cloraria* was found to share a barcode with a dissected male of *C. viridata* from the central Bavarian Danubian valley (the same barcode sequence occurs in Finnish populations of *C. viridata*). The identity of barcodes in this limited sample raises questions about the validity of *C. cloraria* and *C. viridata* or, at the very least, the characters currently employed for their discrimination.

Although this represents the sole case of apparent barcode sharing in the study area, we anticipate that further study may reveal barcode sharing in a few other taxa:

#### *Thera cembrae* (Kitt, 1912) – *Thera obeliscata* (Hübner, 1787)

**Figure 3:**
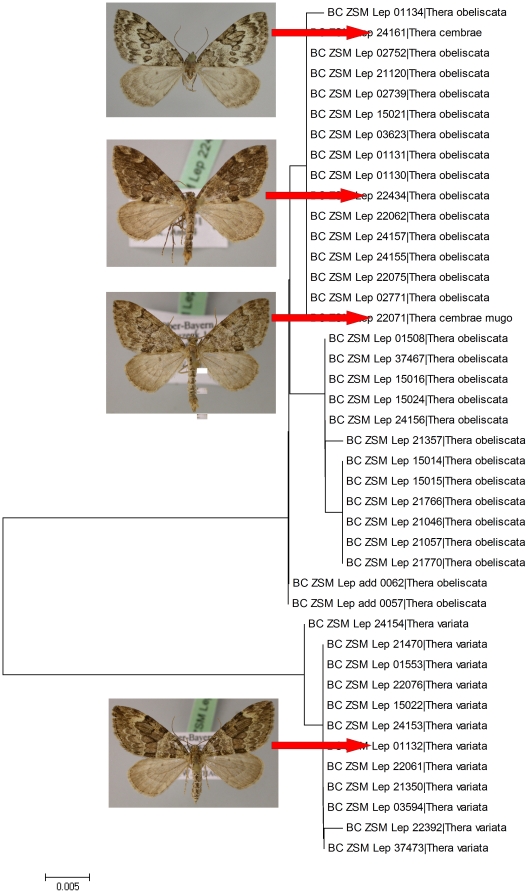
Neighbour joining tree (K2P, fragments of >500 bp), of COI divergences among Bavarian specimens of Thera. Branches with specimen ID-number from BOLD and species name. Both T. cembrae and T. cembrae mugo may be synonyms (‘ecological races’) of Thera obeliscata.

The genus *Thera* is one of the most taxonomically difficult cases in European Lepidoptera. Traditional morphological approaches cannot discriminate all the species reliably and habitus is generally very variable. The two high mountain taxa, *T. cembrae* Kitt, 1912 and *T. mugo* Burmann & Tarmann, 1983, were described based on slight differences in morphology and host-plant use (*Pinus cembra*, *Pinus mugo* respectively). The validity and rank of both taxa have been questioned repeatedly, recently leading to the synonymisation of *T. mugo* with *T. cembrae* [20]. In fact, the male and female genitalia of both *T. cembrae* and *T. mugo* lack any constant differential feature from several congeners. Since both taxa share the same barcode as the lowland species *Thera obeliscata* (Hübner, 1787) (Figure 3) we conclude that they are simply altitudinal forms of the latter species, ‘ecological races’ with peculiar host-plant use. Unless further taxonomic research validates their status as distinct species, *T. cembrae* and *T. obeliscata* cannot be regarded as ‘barcode sharing species’.

#### *Lycia zonaria* (Denis & Schiffermüller, 1775) – *Lycia alpina* (Sulzer, 1776)

This species pair possesses quite clear differences in external appearance coupled with ecological differences as the former is a lowland species, while the latter is restricted to montane settings. Male and female genitalia are similar, but with several constant differential features. As this species pair was found to share barcodes at sites in Austria, Italy (*L. alpina*), France, Hungary and Turkey (*L. zonaria*), Bavarian populations will probably yield the same result. Interestingly, a third, traditionally recognized species, *Lycia graecarius* (Staudinger, 1861) from the Balkan Peninsula shares the same barcode, while the Italian sister species *L. florentina* (Stefanelli, 1882) shows a genetic divergence of 3.4%.

#### *Perizoma affinitata* (Stephens, 1831) – *Perizoma hydrata* (Treitschke, 1829)

These two species show clearly different morphology [17], but possess very similar barcodes in Bavaria as well as in Italy. However, the two species can still be distinguished: *P. affinitata* includes two haplotypes, one differing by just 0.15% (1 base pair) from *P. hydrata*, while the other haplotype differs by 0.71%. Their limited divergence suggests a very recent origin for these taxa or mitochondrial exchange during the Pleistocene. Interestingly, specimens of *P. hydrata* from Turkey have a strikingly different barcode from those of European populations, perhaps representing the original haplotype.

#### *Sciadia* and *Elophos*

Both genera include alpine species, the genus *Sciadia* was recently revised [21]: New data from the whole Alpine arc (n = 41 and 45 barcodes respectively for the two genera) reveal the existence of several genetic lineages within *Sciadia* which often, but not always, correspond to the five clear lineages that are apparent through studies of genital morphology [21]. Cases of barcode sharing involve four of the five species in the genus *Sciadia*: *S. tenebraria* (Esper, 1806), *S. innuptaria* (Herrich-Schäffer, 1852), *S. dolomitica* Huemer & Hausmann, 2009, and *S. slovenica* Leraut, 2008. Nevertheless, all Bavarian vouchers belong to a ‘clean’ cluster of *S. tenebraria* from Bavaria, northern and western Austria with closely related populations in the western Alps, but without barcode-sharing populations from elsewhere. Interestingly, another genetically polymorphic species, *Elophos zelleraria* (Freyer, 1836) includes different haplotypes, which share or almost share their barcodes with the same four *Sciadia* species. Bavarian vouchers of *E. zelleraria* share barcodes with *S. tenebraria* from Austria, Italy and Switzerland. Barcode-sharing in the study area itself was not observed, but it cannot be excluded without further sampling. Another species, *Elophos caelibaria* (Heydenreich, 1851), shares barcodes with two *Sciadia* specimens (*S. tenebraria* and *S. slovenica*) from Italy and Slovenia. In all these cases, sympatric and exact barcode sharing is not yet observed. Of course, our data question the validity of the current taxonomic concept at genus level and suggest to unite all these species pairs with supposed close relationships into one single genus.

#### *Cyclophora punctaria* (Linnaeus, 1758) – *Cyclophora suppunctaria* (Zeller, 1847)

These two species have clear morphological and distributional differences as the latter species is widely distributed in southern Europe, but absent from Bavaria. The two species show little barcode divergence (0.15%), but it seems to enable their unambiguous discrimination (n = 13 and 9 barcodes respectively).

### Possible Cases of Local Introgression

Apart from the case of *Sciadia* and *Elophos* (see above), there are three relatively young species pairs that ordinarily show clear genetic divergence, but where some specimens appear to have ‘the wrong’ mitochondrial genotype, not corresponding to the expectation from their morphological identification. Such cases were detected in three species pairs, all involving taxa where identification through traditional means is challenging so all these cases require further detailed analysis and re-identifications:

#### *Eupithecia orphnata* Petersen, 1909 – *Eupithecia subumbrata* (Denis & Schiffermüller, 1775)

Genetic distance 2.3%, but one barcode of a dissected Bavarian specimen appeared in the wrong cluster. Similar conflicts have been found in countries outside Bavaria. It is important to verify the constancy of the genitalic features that are currently used to diagnose this species pair [17].

#### *Eupithecia pulchellata* Stephens, 1831 *– Eupithecia pyreneata* Mabille, 1871

Genetic distance 4.3%, no conflicts in Bavarian data, but conflicts have been detected in specimens from some other regions. Since identifications based on morphology and genitalia are unreliable, detail studies involving rearing and hybridization studies coupled with DNA barcoding of specimens from verified host-plants should be undertaken.

#### *Isturgia roraria* (Fabricius, 1776) – *Isturgia limbaria* (Fabricius, 1775)

Genetic distance 4.1%, no conflicts in Bavarian data, but conflicts have been detected in specimens from other regions. Prior work has suggested occasional hybridization between these two species in other regions [19].

### Cases of Deep Intraspecific Divergence

**Figure 4:**
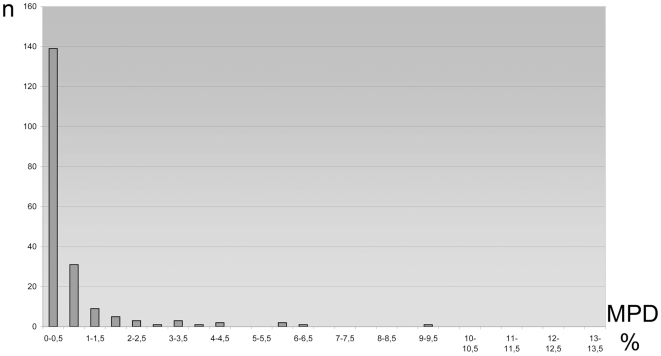
Maximum Pairwise Distances (MPD) for Bavarian geometrid species. Histogram of species numbers (n) by 0.5 per cent-classes. This analysis is based on 658 bp sequences, excludes singletons, and employs a K2P distance model. Species with a MPD >2.0% are discussed in the text.

Most Bavarian geometrids show little intraspecific sequence variation at COI (Figure 4), but approximately 5 per cent of the species were found to include two lineages with a deep split of more than 2 per cent between the component haplotypes. Fourteen ‘traditionally recognized species’ among the 303 species with full-length barcodes (912 individuals, Figure 4) met this condition. When analysis was expanded to the complete data set of 1,321 specimens (>500 bp; 325 species), six other cases of deep divergence were revealed. Eleven of these 20 cases involve a single specimen outside the main clade, but nine taxa included multiple individuals in each barcode cluster. Seven of the splits show more than 4% divergence, and the deepest is 9.2% between the clusters. None of the 20 species showed any obvious correlation between members of different barcode clusters and wing coloration or pattern. Genitalic dissections and sequencing of nuclear genes are underway to ascertain if some or all of these cases represent overlooked species pairs.

Closely related, young species pairs may be overlooked by a 2 per cent screening threshold, but they can still show constant barcode differentiation as emphasized by the example of revised taxonomy of *Eupithecia goossensiata* (see above, Figure 2).

The present analysis showed that mean intraspecific variation was 0.73% (SE = 0.033; n = 2,257 comparisons in the analysis of full-length barcodes) when all species of Bavarian geometrids were included, but this value was elevated by the inclusion of the 20 species with deep barcode divergences, some of which may represent species pairs overlooked by the current taxonomic system. When these taxa are excluded from the analysis, mean intraspecific variation drops to 0.23% (SE = 0.007; n = 1,964 comparisons in the analysis of full-length barcodes). For possible influence of low sample size see discussion.

### Genetic Divergences Correlated with Geography

Most cases of intraspecific divergence involved the sympatric occurrence of both barcode clusters, but sample size must be increased to properly examine geographical patterns within Bavaria. Among several candidate taxa, we give just one example, since larger-scale analysis of most taxa is incomplete.

The two haplotype clusters detected in *Coenotephria salicata* (Hübner, 1799) showed 4.4% sequence divergence. These lineages showed a clear geographical pattern, one was restricted to the eastern Bavarian Alps, while the other was found in the northern mountains of the ‘Bayerischer Wald’. The first cluster included three individuals distinct from all other specimens of *C. salicata* (n = 11) from the Alps, but sharing the barcode of a specimen from the British Isles. Interestingly, the northern Bavarian lineage was closely similar to the barcodes of *C. salicata* from Slovenia, Austria, Switzerland and central Italian mountains (n = 11).

## Discussion

### Identification Success and Young Species

The present study has examined patterns of DNA barcode variation in 400 species of Bavarian geometrids. The technique proved to work efficiently, enabling unambiguous genetic re-identification for at least 96.5 per cent of the fauna. Cases of low sequence divergence (<4%) all involve species pairs known as taxonomically problematic. Low COI distances of 1–2.5 per cent, as observed in eight Bavarian species pairs (Table 1), may correspond to divergence times from a common ancestor of roughly 0.5 to 1 million years [21]–[24] but substitution rates can be elevated over short intervals because of mutational hotspots [25]–[26]. Inter- and postglacial isolation processes due to fragmented distribution areas and restriction to glacial refugia are thought to be a major driving force for younger speciation events in the European fauna [27]. The postulated ages of the glaciation maxima correspond well to the tentative dating of genetic divergences above.

### Hybridization and Introgression

We emphasize that no case of exact barcode-sharing was detected within Bavaria except that involving a sibling species pair in *Chlorissa* and a second case involving three taxa of *Thera* that likely are synonyms. With increasing sample size, we expect that three other cases of barcode-sharing will be discovered involving species in the genera *Lycia*, *Perizoma*, *Sciadia*. Interestingly, at least one member of the species pairs in four of these five cases inhabits alpine habitats, and often both are allopatric vicariants. The species of the genera *Lycia* and *Cyclophora* are known to hybridize readily in nature and in captivity [19] which correlates well with the cases of barcode-sharing in three European species pairs.

Though awaiting corroboration with additional data, there are three apparent species pairs where there is evidence of rare mitochondrial leakage which may either reflect introgression (hybridization) or other mechanisms leading to mitochondrial exchange between species. Two of these cases concern very closely related sister species (genus *Eupithecia*) with low genetic divergences. The case of apparent ‘horizontal exchange’ of mitochondria between species in the genera *Sciadia* and *Elophos* is a very interesting case for subsequent studies.

### Cryptic Diversity

Studies on the Lepidoptera of Europe have now extended for more than 250 years. Our work confirms the validity of almost all Bavarian geometrid species recognized through prior taxonomic work. However, a surprisingly high number of Bavarian geometrid species (20) was found to show deep barcode divergences. Although it is premature to reach conclusions about the biological implications, we expect that some of these cases represent cryptic diversity as emphasized by the case of *Eupithecia absinthiata* and *E. goossensiata* (Figure 2). DNA barcoding studies have similarly revealed overlooked species in North America [11], [28]. At present, the 20 deep divergences in Bavarian geometrids are being addressed by a multivariate analysis including large-scale dissecting, enlarging sample-size with data connected with reliable ecological traits (e.g. natural host-plants), and sequencing additional markers. The detection of so many cases of deep divergence is surprising given the geographical focus on Bavaria and the comparatively low number of specimens sequenced per species with many taxa represented by singletons and doubletons.

### Comparison with the Fauna of Eastern North America

There is an interesting congruence between the results of the Bavarian barcode survey and those on eastern North American Lepidoptera [11]. Intraspecific variation averaged 0.73% in Bavaria (statistically suffering from low sampling size within-species) and 0.43% in North America, and 5% per cent of the species in both regions showed more than 2.0% sequence divergences at COI. Finally, in both regions, very few species shared identical barcode sequences, 0.5% in Bavaria, 0.7% in eastern North America.

### DNA Barcoding as an Efficient Tool for Nature Sciences

Patterns of DNA barcode variation were examined in 400 species of Bavarian geometrids during the first year of the Barcoding Fauna Bavarica (BFB) project, resulting in a comprehensive DNA library available for re-identification, taxonomic spin-offs and other disciplines. All sequences, georeferenced specimen data and images are freely accessible online. We believe that rapid data release will (a) aid validation of data quality and (b) improve identifications. We believe that this study has established that DNA barcoding provides a reliable, quick and very economical method for monitoring the faunistic and taxonomic aspects of the biodiversity of a whole country. Considering the resources devoted to this study and one earlier investigation [11], we conclude that a task force of ten scientists could, within a decade, coordinate the assembly of a DNA barcode reference library for the global Lepidopteran fauna, including the 155,000 described species [29] and the anticipated number of undescribed species which may raise the total up to 500,000 species [30]. Achieving this goal will require the analysis of at least five million specimens, but a single core facility staffed by 20 sequencing technicians and 15 data entry staff could complete the task. The program seems feasible as the species-rich faunas of the southern hemisphere are well represented in major natural history museums and there are taxonomic experts for most of major taxonomic groups. Because it is ideal to have many specialists involved, the overall Lepidoptera barcode effort seeks to involve as many taxonomic experts as possible. In this fashion, the Linnaean vision of knowing all species of the globe may soon become reality. Our next data release will include records for 3,000 African geometrid species testing and showing the suitability of DNA barcoding for tropical faunas (cf [10]).

We are confident that barcode reference libraries will play an important role in biomonitoring programs linked to industrial development, soil and water protection, pest control in forestry and agriculture, food and seed control, environmental studies, nature conservation, performance of monitoring and biodiversity assessment, such as subsequent biological research. Our DNA barcoding studies on Bavarian geometrid moths has not only raised a myriad of biological questions, and further work will provide many answers concerning hostplant specificity, phylogeographic patterns, genetic distances correlated with phenological traits. Several studies have already benefited from the new Bavarian barcode data, such as an ecological study monitoring herbivores on a neophytic plant [31], the discovery of several cryptic Bavarian beetle species (Hendrich, Balke pers. comm.), the revision of a geometrid genus [21] and the detection of three overlooked Microlepidoptera species for the fauna of Bavaria [32], [33]. Additional papers detailing barcode results for Bavarian Lepidoptera, Hymenoptera and Coleoptera are under assembly (Schmidt, Hendrich, Balke, Segerer pers. comm.), and numerous applications in monitoring, land management and environmental research are under investigation.

## Supporting Information

Appendix S1 **Species list and sequencing success.** Species list of Bavarian geometrids, sequencing success, sample size and supplementary material; *  =  submitted, but awaiting analysis; mfl  =  maximum fragment length (number of base pairs); n  =  sample size (number of sequences)
(PDF)

Appendix S2 **Neighbour joining tree of Bavarian geometrids - exemplary data.** Neighbour joining tree (Kimura 2 Parameter) for selected vouchers of Bavarian geometrids, one specimen per species selected (full-fragment analysis with a few additional sequences >600 bp; with species name and specimen ID in BOLD), including 317 species from Bavaria; ‘species’ with deep divergences are only represented by one arbitrarily chosen lineage.
(PDF)

Appendix S3 **GenBank Accession numbers.** List of species name, GenBank Accession numbers, and specimens-ID (from BOLD database) for the Bavarian vouchers with barcodes.
(PDF)
