# Supplementary material for: DNA Barcoding the Geometrid Fauna of Bavaria (Lepidoptera): Successes, Surprises, and Questions
Source: PLoS One. 2011 Feb 14;6(2):e17134. doi: 10.1371/journal.pone.0017134 (PMC3040642; doi:10.1371/journal.pone.0017134)
Supplement: Appendix S1 — Species list and sequencing success. Species list of Bavarian geometrids, sequencing success, sample size and supplementary material; * = submitted, but awaiting analysis; mfl = maximum fragment length (number of base pairs); n = sample size (number of sequences) (PDF) [file pone.0017134.s001.pdf]

SUPPORTING INFORMATION

Appendix S1: Species list and sequencing success

Species list of Bavarian geometrids, sequencing success, sample size and supplementary material; * = submitted and under analysis; mfl = maximum fragment length (number of base pairs); n = sample size (number of sequences)

Archiearinae

Species	mfl	n	origins supplementary material (mfl)
Archiearis parthenias	407	1	
Boudinotiana notha	658	1	

Ennominae

Species	mfl	n	origins supplementary material (mfl)
Abraxas grossulariata	/	0	Finland, Italy, France etc. (658)
Abraxas sylvata	658	1	
Aethalura punctulata	658	4	
Agriopis aurantiaria	658	6	
Agriopis bajaria	616	1	
Agriopis leucophaearia	658	2	
Agriopis marginaria	658	7	
Alcis bastelbergeri	658	1	
Alcis jubata	/	0	Austria, Finland (658)
Alcis repandata	658	15	
Aleucis distinctata	/	0	Bavaria*, France (658)
Alsophila aceraria	/	0	absent from BOLD
Alsophila aescularia	658	5	
Angerona prunaria	658	4	
Apeira syringaria	658	5	
Apocheima hispidaria	658	1	
Apocheima pilosaria	658	3	
Arichanna melanaria	658	2	

Artiora evonymaria	/	0	Austria* (/)
Ascotis selenaria	/	0	Bavaria*, Italy, France etc. (658)
Aspitates gilvaria	658	2	
Biston betularia	658	8	
Biston strataria	658	4	
Bupalus piniaria	658	2	
Cabera exanthemata	658	11	
Cabera leptographa	/	0	Poland* (/)
Cabera pusaria	658	6	
Campaea honoraria	/	0	Italy (658)
Campaea margaritaria	658	5	
Cepphis advenaria	658	5	
Charissa ambiguata	658	2	
Charissa glaucinaria	658	3	
Charissa intermedia	565	1	
Charissa obscurata	658	2	
Charissa pullata	658	1	
Chiasmia clathrata	658	4	
Cleora cinctaria	658	1	
Cleorodes lichenaria	/	0	Italy, Finland (658)
Colotois pennaria	658	6	
Crocallis elinguaria	658	6	
Crocallis tusciaria	/	0	Italy, Hungary, Greece etc. (658)
Deileptenia ribeata	658	5	
Ectropis crepuscularia	658	39	
Elophos caelibaria	658	1	
Elophos dilucidaria	658	6	
Elophos serotinaria	/	0	Austria (658)
Elophos vittaria	658	1	
Elophos zelleraria	609	2	
Ematurga atomaria	618	2	

Ennomos alniaria	658	2	
Ennomos autumnaria	658	2	
Ennomos erosaria	658	6	
Ennomos fuscantaria	/	0	Bavaria*, Italy, Finland (658)
Ennomos quercinaria	658	5	
Epione repandaria	658	4	
Epione vespertaria	658	1	
Epirranthis diversata	/	0	Finland (658)
Erannis defoliaria	658	9	
Fagivorina arenaria	658	1	
Glacies alpinata	658	2	
Glacies canaliculata	658	1	
Glacies coracina	658	1	
Glacies noricana	658	1	
Gnophos furvata	658	2	
Gnophos obfuscata	658	5	
Heliomata glarearia	/	0	Bavaria*, France, Italy etc. (658)
Hylaea fasciaria	658	10	
Hypomecis punctinalis	658	6	
Hypomecis roboraria	658	4	
Hypoxystis pluviaria	658	3	
Isturgia limbaria	658	2	
Isturgia murinaria	/	0	France, Bosnia, Turkey etc. (658)
Isturgia roraria	658	1	
Ligdia adustata	658	8	
Lomaspilis marginata	658	2	
Lomographa bimaculata	658	5	
Lomographa temerata	658	3	
Lycia alpina	/	0	Austria, Italy (658)
Lycia hirtaria	658	12	
Lycia isabellae	/	0	Austria (407)

Lycia pomonaria	/	0	Finland (658)
Lycia zonaria	/	0	France, Hungary, Turkey (658)
Macaria alternata	658	2	
Macaria artesiaria	658	2	
Macaria brunneata	658	4	
Macaria fusca	532	1	
Macaria liturata	658	6	
Macaria notata	658	8	
Macaria signaria	658	4	
Macaria wauaria	658	5	
Odontopera bidentata	658	5	
Opisthograptis luteolata	658	3	
Ourapteryx sambucaria	658	2	
Pachycnemia hippocastanaria	658	2	
Paradarisa consonaria	658	3	
Parectropis similaria	658	3	
Perconia strigillaria	/	0	Italy, Finland (658)
Peribatodes rhomboidaria	658	10	
Peribatodes secundaria	658	4	
Petrophora chlorosata	658	4	
Plagodis dolabraria	658	4	
Plagodis pulveraria	658	6	
Pseudopanthera macularia	658	2	
Psodos quadrifaria	658	2	
Pungeleria capreolaria	658	2	
Sciadia tenebraria	658	4	
Selenia dentaria	658	3	
Selenia lunularia	658	3	
Selenia tetralunaria	658	5	
Selidosema brunnearia	/	0	Germany, Italy, Greece etc. (658)
Siona lineata	658	4	

Stegania cararia	/	0	Bavaria* (/)
Stegania trimaculata	658	1	
Synopsia sociaria	/	0	Italy, Turkey (658)
Tephronia sepiaria	/	0	France, Italy, Spain etc. (658)
Theria primaria	658	2	
Theria rupicapraria	658	2	

Geometrinae

Species	mfl	n	origins supplementary material (mfl)
Aplasta ononaria	/	0	Italy*, Israel (658)
Chlorissa cloraria	586	1	
Chlorissa viridata	658	1	
Comibaena bajularia	658	4	
Geometra papilionaria	658	3	
Hemistola chrysoprasaria	658	5	
Hemithea aestivaria	658	4	
Jodis lactearia	658	3	
Jodis putata	658	1	
Pseudoterpna pruinata	658	1	
Thalera fimbrialis	658	3	
Thetidia smaragdaria	658	3	

Larentiinae

Species	mfl	n	origins supplementary material (mfl)
Acasis appensata	/	0	Austria, Finland, Italy (658)
Acasis viretata	658	4	
Anticlea badiata	658	6	
Anticlea derivata	658	3	
Anticollix sparsata	658	4	
Aplocera efformata	658	5	
Aplocera plagiata	658	1	
Aplocera praeformata	658	3	

Asthena albulata	658	2	
Asthena anseraria	658	3	
Baptria tibiale	658	1	
Camptogramma bilineata	658	6	
Carsia sororiata	658	1	
Cataclysme riguata	288	2	
Catarhoe cuculata	658	3	
Catarhoe rubidata	658	2	
Chesias legatella	548	2	
Chesias rufata	/	0	Italy, Spain (658)
Chloroclysta miata	252	1	
Chloroclysta siterata	658	19	
Chloroclystis v-ata	658	7	
Cidaria fulvata	658	6	
Coenocalpe lapidata	/	0	Finland, Turkey (658)
Coenotephria salicata	658	4	
Coenotephria tophaceata	658	2	
Colostygia aptata	658	4	
Colostygia aqueata	633	1	
Colostygia austriacaria	/	0	Austria, Italy (658)
Colostygia kollariaria	658	1	
Colostygia multistrigaria	/	0	Germany, Italy (658)
Colostygia olivata	658	6	
Colostygia pectinataria	658	3	
Colostygia puengeleri	/	0	Bavaria*, Austria, Switzerland (658)
Colostygia turbata	658	2	
Cosmorhoe ocellata	658	3	
Costaconvexa polygrammata	/	0	Italy, Turkey, Israel (658)
Dysstroma citrata	658	3	
Dysstroma truncata	658	18	
Ecliptopera capitata	658	6	

Ecliptopera silaceata	658	10	
Electrophaes corylata	658	7	
Entephria caesiata	658	7	
Entephria cyanata	658	2	
Entephria flavata	658	2	
Entephria flavicinctata	658	1	
Entephria infidaria	658	1	
Entephria nobiliaria	658	2	
Epirrhoe alternata	658	10	
Epirrhoe galiata	658	4	
Epirrhoe hastulata	594	2	
Epirrhoe molluginata	658	4	
Epirrhoe pupillata	/	0	Finland, China (658)
Epirrhoe rivata	658	2	
Epirrhoe tristata	658	5	
Epirrita autumnata	658	7	
Epirrita christyi	658	17	
Epirrita dilutata	626	4	
Euchoeca nebulata	658	4	
Eulithis mellinata	/	0	Bavaria*, Finland (658)
Eulithis populata	658	4	
Eulithis prunata	658	6	
Eulithis testata	/	0	Bavaria*, Finland, Canada (658)
Euphyia adumbraria	654	1	
Euphyia biangulata	654	1	
Euphyia frustata	657	1	
Euphyia scripturata	658	1	
Euphyia unangulata	658	2	
Eupithecia abbreviata	658	3	
Eupithecia abietaria	658	3	
Eupithecia absinthiata	658	12	

Eupithecia actaeata	658	2	
Eupithecia analoga	632	1	
Eupithecia assimilata	658	8	
Eupithecia cauchiata	658	3	
Eupithecia centaureata	658	2	
Eupithecia conterminata	658	3	
Eupithecia denotata	658	2	
Eupithecia denticulata	/	0	Turkey (601)
Eupithecia distinctaria	658	4	
Eupithecia dodoneata	658	6	
Eupithecia egenaria	658	2	
Eupithecia exiguata	658	9	
Eupithecia expallidata	658	2	
Eupithecia extraversaria	658	1	
Eupithecia extremata	/	0	Turkey, Israel (658)
Eupithecia goossensiata	658	3	
Eupithecia haworthiata	658	5	
Eupithecia icterata	658	6	
Eupithecia immundata	658	2	
Eupithecia impurata	658	2	
Eupithecia indigata	658	4	
Eupithecia innotata	615	2	
Eupithecia insigniata	658	1	
Eupithecia intricata	658	11	
Eupithecia inturbata	658	10	
Eupithecia irriguata	/	0	Finland, Israel, Lebanon (658)
Eupithecia lanceata	658	6	
Eupithecia laquaearia	/	0	Bavaria*, Italy (658)
Eupithecia lariciata	658	4	
Eupithecia linariata	658	2	
Eupithecia millefoliata	/	0	Bavaria*, Finland (658)

Eupithecia nanata	658	4	
Eupithecia ochridata	/	0	Germany, Italy, Bulgaria (658)
Eupithecia orphnata	658	1	
Eupithecia pimpinellata	641	2	
Eupithecia plumbeolata	658	4	
Eupithecia pulchellata	658	1	
Eupithecia pusillata	658	4	
Eupithecia pygmaeata	/	0	Finland (658)
Eupithecia pyreneata	581	2	
Eupithecia satyrata	658	4	
Eupithecia selinata	658	3	
Eupithecia semigraphata	658	5	
Eupithecia silenata	658	1	
Eupithecia simpliciata	/	0	Finland (658)
Eupithecia sinuosaria	658	1	
Eupithecia subfuscata	658	26	
Eupithecia subumbrata	658	4	
Eupithecia succenturiata	240	1	
Eupithecia tantillaria	658	15	
Eupithecia tenuiata	658	5	
Eupithecia tripunctaria	658	14	
Eupithecia trisignaria	658	2	
Eupithecia undata	/	0	Italy (658)
Eupithecia valerianata	658	3	
Eupithecia venosata	658	3	
Eupithecia veratraria	658	6	
Eupithecia virgaureata	658	20	
Eupithecia vulgata	658	9	
Eustroma reticulata	658	6	
Gagitodes sagittata	658	3	
Gandaritis pyraliata	658	3	

Gymnoscelis rufifasciata	658	7	
Horisme aemulata	658	2	
Horisme aquata	/	0	Mongolia, China, Russia* (658)
Horisme corticata	/	0	Turkey, Greece (609)
Horisme radicaria	658	4	
Horisme tersata	658	2	
Horisme vitalbata	658	3	
Hydrelia flammeolaria	658	4	
Hydrelia sylvata	658	3	
Hydriomena furcata	658	4	
Hydriomena impluviata	658	5	
Hydriomena ruberata	658	3	
Lampropteryx otregiata	658	4	
Lampropteryx suffumata	658	4	
Larentia clavaria	/	0	Denmark, Italy, Finland etc. (658)
Lithostege farinata	/	0	Greece, Austria, Russia* (658)
Lithostege griseata	/	0	U.K., Russia, Ukraine (658)
Lobophora halterata	658	10	
Lythria cruentaria	/	0	Finland, Italy* (641)
Lythria plumularia	/	0	Switzerland (607)
Lythria purpuraria	/	0	Turkey, Iran, Italy*, Greece* (658)
Martania taeniata	658	2	
Melanthia alaudaria	658	1	
Melanthia procellata	658	8	
Mesoleuca albicillata	658	5	
Mesotype didymata	658	2	
Mesotype parallelolineata	577	2	
Mesotype verberata	658	5	
Minoa murinata	648	3	
Nebula achromaria	/	0	Austria, Italy (658)
Nebula nebulata	658	3	

Nothocasis sertata	658	7	
Odezia atrata	658	4	
Operophtera brumata	658	12	
Operophtera fagata	658	3	
Orthonama obstipata	658	4	
Orthonama vittata	658	5	
Pareulype berberata	658	5	
Pasiphila chloerata	658	3	
Pasiphila debiliata	658	1	
Pasiphila rectangulata	658	13	
Pelurga comitata	624	1	
Pennithera firmata	658	7	
Perizoma affinitata	658	3	
Perizoma albulata	658	3	
Perizoma alchemillata	658	16	
Perizoma bifaciata	615	3	
Perizoma blandiata	658	3	
Perizoma flavofasciata	658	4	
Perizoma hydrata	658	1	
Perizoma incultaria	658	1	
Perizoma lugdunaria	/	0	Bavaria* (/)
Perizoma minorata	658	3	
Perizoma obsoletata	658	2	
Phibalapteryx virgata	658	3	
Philereme transversata	658	2	
Philereme vetulata	658	5	
Plemyria rubiginata	658	1	
Pterapherapteryx sexalata	658	2	
Rheumaptera cervinalis	658	7	
Rheumaptera hastata	658	3	
Rheumaptera subhastata	/	0	Bavaria*, Finland (658)

Rheumaptera undulata	658	3	
Scotopteryx bipunctaria	658	2	
Scotopteryx chenopodiata	658	4	
Scotopteryx coarctaria	/	0	Turkey, Italy* (658)
Scotopteryx luridata	658	2	
Scotopteryx moeniata	658	3	
Scotopteryx mucronata	/	0	Bavaria*, Turkey, Italy* etc. (612)
Spargania luctuata	658	2	
Thera britannica	658	3	
Thera cembrae	658	1	
Thera cognata	658	2	
Thera juniperata	658	4	
Thera obeliscata	658	28	
Thera variata	658	13	
Thera vetustata	658	1	
Trichopteryx carpinata	658	4	
Trichopteryx polycommata	658	5	
Triphosa dubitata	658	4	
Triphosa sabaudiata	658	2	
Venusia blomeri	658	4	
Venusia cambrica	658	1	
Xanthorhoe biriviata	658	6	
Xanthorhoe decoloraria	658	1	
Xanthorhoe designata	658	5	
Xanthorhoe ferrugata	658	13	
Xanthorhoe fluctuata	658	12	
Xanthorhoe incursata	658	1	
Xanthorhoe montanata	658	5	
Xanthorhoe quadrifasiata	658	7	
Xanthorhoe spadicearia	658	8	

Sterrhinae

Species	mfl	n	origins supplementary material (mfl)
Cyclophora albipunctata	658	2	
Cyclophora annularia	636	1	
Cyclophora linearia	658	2	
Cyclophora pendularia	475	1	
Cyclophora porata	/	0	Italy, Spain (658)
Cyclophora punctaria	658	4	
Cyclophora puppillaria	/	0	Italy, Spain, Turkey etc. (658)
Cyclophora quercimontaria	/	0	Bavaria*, Italy*, Turkey* (/)
Cyclophora ruficiliaria	/	0	Bavaria*, Italy (658)
Idaea aureolaria	658	1	
Idaea aversata	658	4	
Idaea biselata	658	5	
Idaea contiguaria	/	0	absent from BOLD
Idaea degeneraria	/	0	Bavaria*, Italy, Turkey etc. (658)
Idaea deversaria	/	0	Italy, Spain, Finland etc. (658)
Idaea dilutaria	658	1	
Idaea dimidiata	658	4	
Idaea emarginata	658	3	
Idaea fuscovenosa	658	3	
Idaea humiliata	658	2	
Idaea inquinata	658	6	
Idaea laevigata	/	0	Italy, Spain* (633)
Idaea moniliata	/	0	Bavaria*, Italy, Turkey etc. (658)
Idaea muricata	658	2	
Idaea ochrata	/	0	Bavaria*, Italy, Greece etc. (658)
Idaea pallidata	417	2	
Idaea rufaria	/	0	Bavaria*, Italy, Iran (658)
Idaea rusticata	/	0	Italy, Iran (658)
Idaea seriata	658	6	

Idaea serpentata	658	2	
Idaea straminata	658	5	
Idaea subsericeata	/	0	Italy, Turkey etc. (658)
Idaea sylvestraria	658	5	
Idaea trigeminata	/	0	Italy, Greece, Turkey etc. (658)
Rhodometra sacraria	/	0	Italy, Greece, Turkey etc. (658)
Rhodostrophia vibicaria	658	3	
Scopula caricaria	/	0	France, Finland (658)
Scopula decorata	/	0	Italy, Portugal, Turkey etc. (658)
Scopula floslactata	658	6	
Scopula immorata	658	4	
Scopula immutata	658	2	
Scopula incanata	658	2	
Scopula marginepunctata	658	2	
Scopula nemoraria	/	0	absent from BOLD
Scopula nigropunctata	658	4	
Scopula ornata	658	5	
Scopula rubiginata	658	4	
Scopula subpunctaria	658	1	
Scopula ternata	658	1	
Scopula umbelaria	658	1	
Scopula virgulata	/	0	Bosnia, China, Mongolia (658)
Timandra comae	658	7	

Species total: 407

Species with sequences: 400

Species with barcodes (>500 bp): 399

Species with full fragments (658 bp): 393

Bavarian vouchers with sequences:1395 (331 species)

Bavarian vouchers with barcodes (>500 bp): 1321 (325 species)

Bavarian vouchers with full fragments (658 bp): 912 (303 species)
