# Supplementary material for: DNA Barcoding the Geometrid Fauna of Bavaria (Lepidoptera): Successes, Surprises, and Questions
Source: PLoS One. 2011 Feb 14;6(2):e17134. doi: 10.1371/journal.pone.0017134 (PMC3040642; doi:10.1371/journal.pone.0017134)
Supplement: Appendix S2 — Neighbour joining tree of Bavarian geometrids - exemplary data. Neighbour joining tree (Kimura 2 Parameter) for selected vouchers of Bavarian geometrids, one specimen per species selected (full-fragment analysis with a few additional sequences >600 bp; with species name and specimen ID in BOLD), including 317 species from Bavaria; ‘species’ with deep divergences are only represented by one arbitrarily chosen lineage. (PDF) [file pone.0017134.s002.pdf]

Appendix S2: Neighbour joining tree of Bavarian geometrids - exemplary data

Neighbour joining tree (Kimura 2 Parameter) for selected vouchers of Bavarian geometrids, one specimen per species selected (full-fragment analysis with a few additional sequences > 600 bp; with species name and specimen ID in BOLD), including 317 species from the territory of Bavaria; ‘species’ with deep divergences are represented by one arbitrarily chosen lineage only.

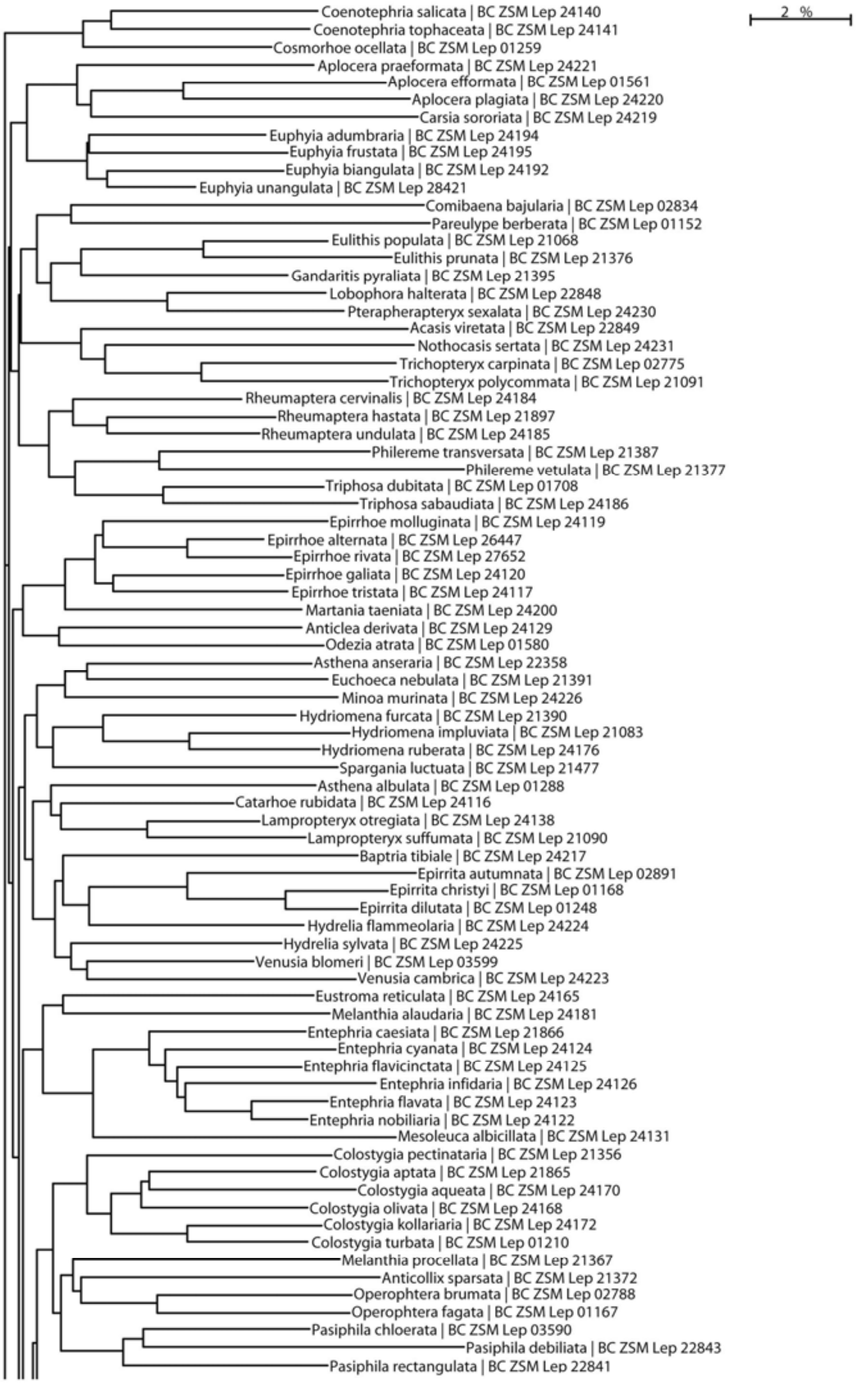

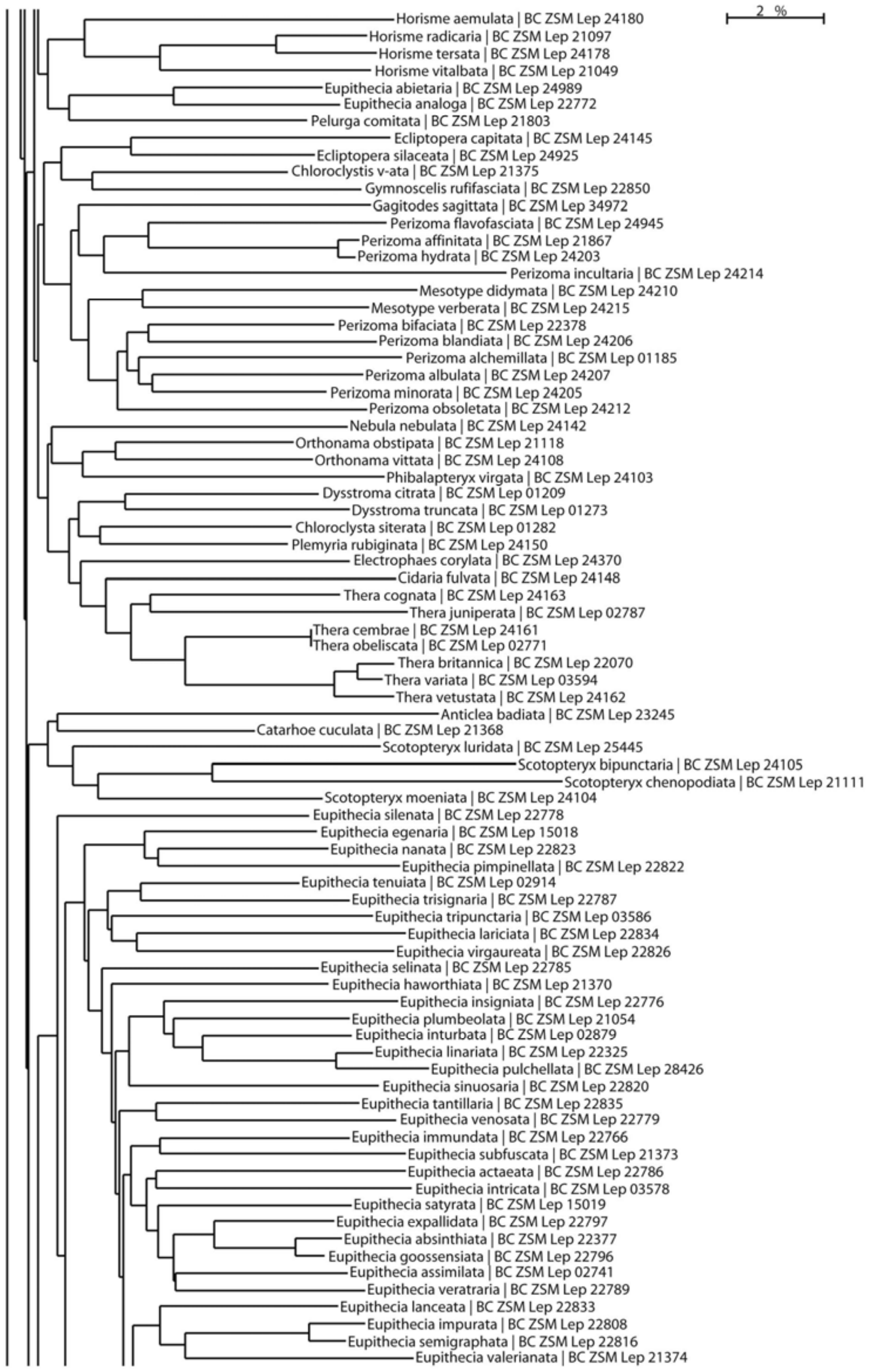
2 %
Horisme aemulata | BC ZSM Lep 24180
Horisme radicaria | BC ZSM Lep 21097
Horisme tersata | BC ZSM Lep 24178
Horisme vitalbata | BC ZSM Lep 21049
Eupithecia abietaria | BC ZSM Lep 24989
Eupithecia analoga | BC ZSM Lep 22772
Pelurga comitata | BC ZSM Lep 21803
Ecliptopera capitata | BC ZSM Lep 24145
Ecliptopera silaceata | BC ZSM Lep 24925
Chloroclystis v-ata | BC ZSM Lep 21375
Gymnoscelis rufifasciata | BC ZSM Lep 22850
Gagitodes sagittata | BC ZSM Lep 34972
Perizoma flavofasciata | BC ZSM Lep 24945
Perizoma affinitata | BC ZSM Lep 21867
Perizoma hydrata | BC ZSM Lep 24203
Perizoma incultaria | BC ZSM Lep 24214
Mesotype didymata | BC ZSM Lep 24210
Mesotype verberata | BC ZSM Lep 24215
Perizoma bifaciata | BC ZSM Lep 22378
Perizoma blandiata | BC ZSM Lep 24206
Perizoma alchemillata | BC ZSM Lep 01185
Perizoma albulata | BC ZSM Lep 24207
Perizoma minorata | BC ZSM Lep 24205
Perizoma obsoletata | BC ZSM Lep 24212
Nebula nebulata | BC ZSM Lep 24142
Orthonama obstipata | BC ZSM Lep 21118
Orthonama vittata | BC ZSM Lep 24108
Phibalapteryx virgata | BC ZSM Lep 24103
Dysstroma citrata | BC ZSM Lep 01209
Dysstroma truncata | BC ZSM Lep 01273
Chloroclysta siterata | BC ZSM Lep 01282
Plemyria rubiginata | BC ZSM Lep 24150
Electrophaes corylata | BC ZSM Lep 24370
Cidaria fulvata | BC ZSM Lep 24148
Thera cognata | BC ZSM Lep 24163
Thera juniperata | BC ZSM Lep 02787
Thera cembrae | BC ZSM Lep 24161
Thera obeliscata | BC ZSM Lep 02771
Thera britannica | BC ZSM Lep 22070
Thera variata | BC ZSM Lep 03594
Thera vetustata | BC ZSM Lep 24162
Anticlea badiata | BC ZSM Lep 23245
Catarhoe cuculata | BC ZSM Lep 21368
Scotopteryx luridata | BC ZSM Lep 25445
Scotopteryx bipunctaria | BC ZSM Lep 24105
Scotopteryx chenopodiata | BC ZSM Lep 21111
Scotopteryx moeniata | BC ZSM Lep 24104
Eupithecia silenata | BC ZSM Lep 22778
Eupithecia egenaria | BC ZSM Lep 15018
Eupithecia nanata | BC ZSM Lep 22823
Eupithecia pimpinellata | BC ZSM Lep 22822
Eupithecia tenuiata | BC ZSM Lep 02914
Eupithecia trisignaria | BC ZSM Lep 22787
Eupithecia tripunctaria | BC ZSM Lep 03586
Eupithecia lariciata | BC ZSM Lep 22834
Eupithecia virgaureata | BC ZSM Lep 22826
Eupithecia selinata | BC ZSM Lep 22785
Eupithecia haworthiata | BC ZSM Lep 21370
Eupithecia insigniata | BC ZSM Lep 22776
Eupithecia plumbeolata | BC ZSM Lep 21054
Eupithecia inturbata | BC ZSM Lep 02879
Eupithecia linariata | BC ZSM Lep 22325
Eupithecia pulchellata | BC ZSM Lep 28426
Eupithecia sinuosaria | BC ZSM Lep 22820
Eupithecia tantillaria | BC ZSM Lep 22835
Eupithecia venosata | BC ZSM Lep 22779
Eupithecia immundata | BC ZSM Lep 22766
Eupithecia subfuscata | BC ZSM Lep 21373
Eupithecia actaeata | BC ZSM Lep 22786
Eupithecia intricata | BC ZSM Lep 03578
Eupithecia satyrata | BC ZSM Lep 15019
Eupithecia expallidata | BC ZSM Lep 22797
Eupithecia absinthiata | BC ZSM Lep 22377
Eupithecia goossensiata | BC ZSM Lep 22796
Eupithecia assimilata | BC ZSM Lep 02741
Eupithecia veratraria | BC ZSM Lep 22789
Eupithecia lanceata | BC ZSM Lep 22833
Eupithecia impurata | BC ZSM Lep 22808
Eupithecia semigraphata | BC ZSM Lep 22816
Eupithecia valerianata | BC ZSM Lep 21374

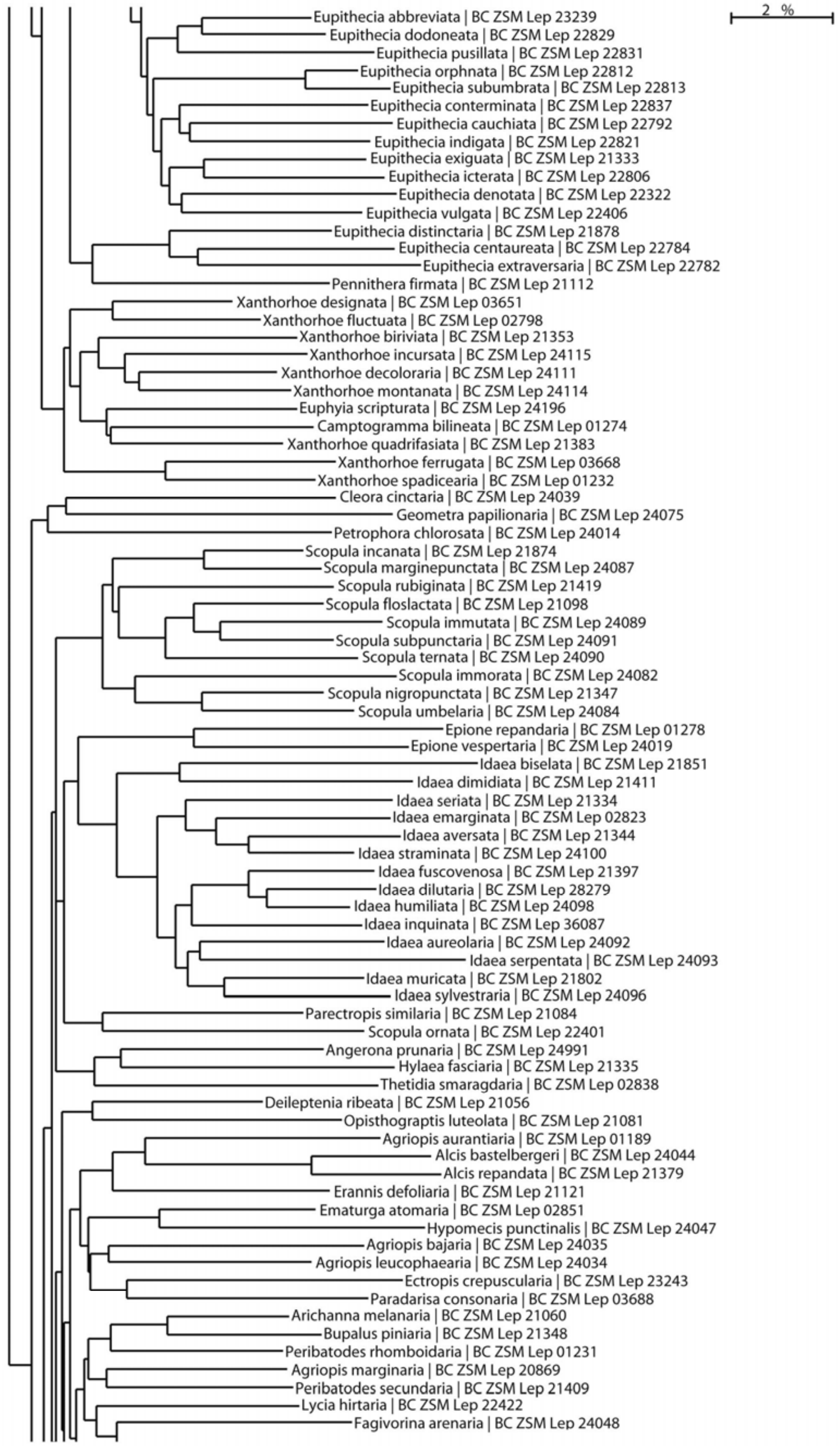
2 %
Eupithecia abbreviata | BC ZSM Lep 23239
Eupithecia dodoneata | BC ZSM Lep 22829
Eupithecia pusillata | BC ZSM Lep 22831
Eupithecia orphnata | BC ZSM Lep 22812
Eupithecia subumbrata | BC ZSM Lep 22813
Eupithecia conterminata | BC ZSM Lep 22837
Eupithecia cauchiata | BC ZSM Lep 22792
Eupithecia indigata | BC ZSM Lep 22821
Eupithecia exiguata | BC ZSM Lep 21333
Eupithecia icterata | BC ZSM Lep 22806
Eupithecia denotata | BC ZSM Lep 22322
Eupithecia vulgata | BC ZSM Lep 22406
Eupithecia distinctaria | BC ZSM Lep 21878
Eupithecia centaureata | BC ZSM Lep 22784
Eupithecia extraversaria | BC ZSM Lep 22782
Pennithera firmata | BC ZSM Lep 21112
Xanthorhoe designata | BC ZSM Lep 03651
Xanthorhoe fluctuata | BC ZSM Lep 02798
Xanthorhoe biriviata | BC ZSM Lep 21353
Xanthorhoe incursata | BC ZSM Lep 24115
Xanthorhoe decoloraria | BC ZSM Lep 24111
Xanthorhoe montanata | BC ZSM Lep 24114
Euphyia scripturata | BC ZSM Lep 24196
Camptogramma bilineata | BC ZSM Lep 01274
Xanthorhoe quadrifasiata | BC ZSM Lep 21383
Xanthorhoe ferrugata | BC ZSM Lep 03668
Xanthorhoe spadicearia | BC ZSM Lep 01232
Cleora cinctaria | BC ZSM Lep 24039
Geometra papilionaria | BC ZSM Lep 24075
Petrophora chlorosata | BC ZSM Lep 24014
Scopula incanata | BC ZSM Lep 21874
Scopula marginepunctata | BC ZSM Lep 24087
Scopula rubiginata | BC ZSM Lep 21419
Scopula floslactata | BC ZSM Lep 21098
Scopula immutata | BC ZSM Lep 24089
Scopula subpunctaria | BC ZSM Lep 24091
Scopula ternata | BC ZSM Lep 24090
Scopula immorata | BC ZSM Lep 24082
Scopula nigropunctata | BC ZSM Lep 21347
Scopula umbelaria | BC ZSM Lep 24084
Epione repandaria | BC ZSM Lep 01278
Epione vespertaria | BC ZSM Lep 24019
Idaea biselata | BC ZSM Lep 21851
Idaea dimidiata | BC ZSM Lep 21411
Idaea seriata | BC ZSM Lep 21334
Idaea emarginata | BC ZSM Lep 02823
Idaea aversata | BC ZSM Lep 21344
Idaea straminata | BC ZSM Lep 24100
Idaea fuscovenosa | BC ZSM Lep 21397
Idaea dilutaria | BC ZSM Lep 28279
Idaea humiliata | BC ZSM Lep 24098
Idaea inquinata | BC ZSM Lep 36087
Idaea aureolaria | BC ZSM Lep 24092
Idaea serpentata | BC ZSM Lep 24093
Idaea muricata | BC ZSM Lep 21802
Idaea sylvestraria | BC ZSM Lep 24096
Parectropis similaria | BC ZSM Lep 21084
Scopula ornata | BC ZSM Lep 22401
Angerona prunaria | BC ZSM Lep 24991
Hylaea fasciaria | BC ZSM Lep 21335
Thetidia smaragdaria | BC ZSM Lep 02838
Deileptenia ribeata | BC ZSM Lep 21056
Opisthograptis luteolata | BC ZSM Lep 21081
Agriopis aurantiaria | BC ZSM Lep 01189
Alcis bastelbergeri | BC ZSM Lep 24044
Alcis repandata | BC ZSM Lep 21379
Erannis defoliaria | BC ZSM Lep 21121
Ematurga atomaria | BC ZSM Lep 02851
Hypomecis punctinalis | BC ZSM Lep 24047
Agriopis bajaria | BC ZSM Lep 24035
Agriopis leucophaearia | BC ZSM Lep 24034
Ectropis crepuscularia | BC ZSM Lep 23243
Paradarisa consonaria | BC ZSM Lep 03688
Arichanna melanaria | BC ZSM Lep 21060
Bupalus piniaria | BC ZSM Lep 21348
Peribatodes rhomboidaria | BC ZSM Lep 01231
Agriopis marginaria | BC ZSM Lep 20869
Peribatodes secundaria | BC ZSM Lep 21409
Lycia hirtaria | BC ZSM Lep 22422
Fagivorina arenaria | BC ZSM Lep 24048

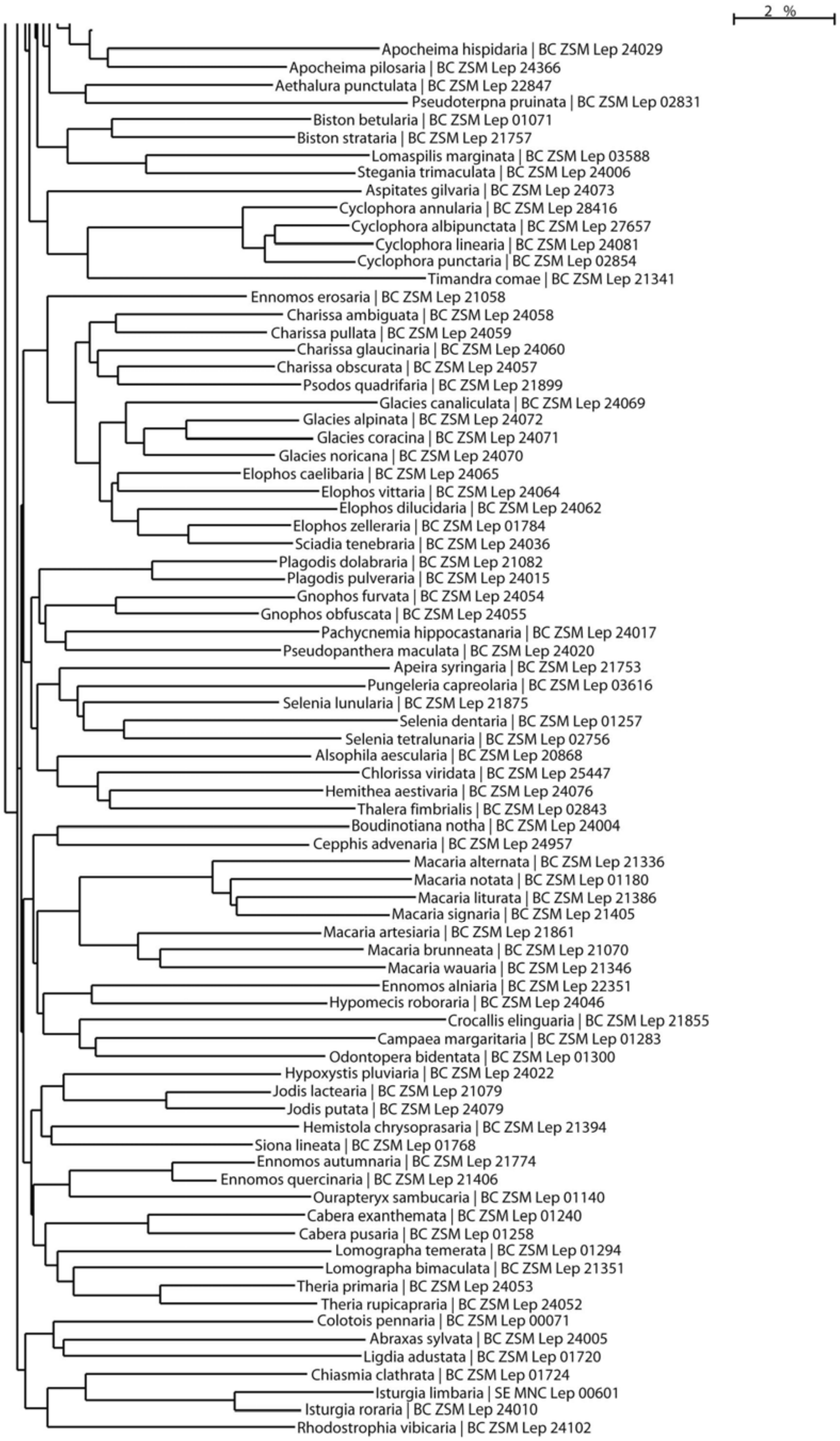
2 %
Apocheima hispidaria | BC ZSM Lep 24029
Apocheima pilosaria | BC ZSM Lep 24366
Aethalura punctulata | BC ZSM Lep 22847
Pseudoterpna pruinata | BC ZSM Lep 02831
Biston betularia | BC ZSM Lep 01071
Biston strataria | BC ZSM Lep 21757
Lomaspilis marginata | BC ZSM Lep 03588
Stegania trimaculata | BC ZSM Lep 24006
Aspitates gilvaria | BC ZSM Lep 24073
Cyclophora annularia | BC ZSM Lep 28416
Cyclophora albipunctata | BC ZSM Lep 27657
Cyclophora linearia | BC ZSM Lep 24081
Cyclophora punctaria | BC ZSM Lep 02854
Timandra comae | BC ZSM Lep 21341
Ennomos erosaria | BC ZSM Lep 21058
Charissa ambiguata | BC ZSM Lep 24058
Charissa pullata | BC ZSM Lep 24059
Charissa glaucinaria | BC ZSM Lep 24060
Charissa obscurata | BC ZSM Lep 24057
Psodos quadrifaria | BC ZSM Lep 21899
Glacies canaliculata | BC ZSM Lep 24069
Glacies alpinata | BC ZSM Lep 24072
Glacies coracina | BC ZSM Lep 24071
Glacies noricana | BC ZSM Lep 24070
Elophos caelibaria | BC ZSM Lep 24065
Elophos vittaria | BC ZSM Lep 24064
Elophos dilucidaria | BC ZSM Lep 24062
Elophos zelleraria | BC ZSM Lep 01784
Sciadia tenebraria | BC ZSM Lep 24036
Plagodis dolabraria | BC ZSM Lep 21082
Plagodis pulveraria | BC ZSM Lep 24015
Gnophos furvata | BC ZSM Lep 24054
Gnophos obfuscata | BC ZSM Lep 24055
Pachycnemia hippocastanaria | BC ZSM Lep 24017
Pseudopanthera maculata | BC ZSM Lep 24020
Apeira syringaria | BC ZSM Lep 21753
Pungeleria capreolaria | BC ZSM Lep 03616
Selenia lunularia | BC ZSM Lep 21875
Selenia dentaria | BC ZSM Lep 01257
Selenia tetralunaria | BC ZSM Lep 02756
Alsophila aescularia | BC ZSM Lep 20868
Chlorissa viridata | BC ZSM Lep 25447
Hemithea aestivaria | BC ZSM Lep 24076
Thalera fimbrialis | BC ZSM Lep 02843
Boudinotiana notha | BC ZSM Lep 24004
Cepphis advenaria | BC ZSM Lep 24957
Macaria alternata | BC ZSM Lep 21336
Macaria notata | BC ZSM Lep 01180
Macaria liturata | BC ZSM Lep 21386
Macaria signaria | BC ZSM Lep 21405
Macaria artesiaria | BC ZSM Lep 21861
Macaria brunneata | BC ZSM Lep 21070
Macaria wauaria | BC ZSM Lep 21346
Ennomos alniaria | BC ZSM Lep 22351
Hypomecis roboraria | BC ZSM Lep 24046
Crocallis elinguaria | BC ZSM Lep 21855
Campaea margaritaria | BC ZSM Lep 01283
Odontopera bidentata | BC ZSM Lep 01300
Hypoxystis pluviaria | BC ZSM Lep 24022
Jodis lactearia | BC ZSM Lep 21079
Jodis putata | BC ZSM Lep 24079
Hemistola chrysoprasaria | BC ZSM Lep 21394
Siona lineata | BC ZSM Lep 01768
Ennomos autumnaria | BC ZSM Lep 21774
Ennomos quercinaria | BC ZSM Lep 21406
Ourapteryx sambucaria | BC ZSM Lep 01140
Cabera exanthemata | BC ZSM Lep 01240
Cabera pusaria | BC ZSM Lep 01258
Lomographa temerata | BC ZSM Lep 01294
Lomographa bimaculata | BC ZSM Lep 21351
Theria primaria | BC ZSM Lep 24053
Theria rupicapraria | BC ZSM Lep 24052
Colotois pennaria | BC ZSM Lep 00071
Abraxas sylvata | BC ZSM Lep 24005
Ligdia adustata | BC ZSM Lep 01720
Chiasmia clathrata | BC ZSM Lep 01724
Isturgia limbaria | SE MNC Lep 00601
Isturgia roraria | BC ZSM Lep 24010
Rhodostrophia vibicaria | BC ZSM Lep 24102
