# Supplementary material for: DNA Barcoding the Geometrid Fauna of Bavaria (Lepidoptera): Successes, Surprises, and Questions
Source: PLoS One. 2011 Feb 14;6(2):e17134. doi: 10.1371/journal.pone.0017134 (PMC3040642; doi:10.1371/journal.pone.0017134)
Supplement: Appendix S3 — GenBank Accession numbers. List of species name, GenBank Accession numbers, and specimens-ID (from BOLD database) for the Bavarian vouchers with barcodes. (PDF) [file pone.0017134.s003.pdf]

Appendix S3: GenBank Accession numbers

List of species name, GenBank Accession numbers, and specimens-ID (from BOLD database) for the Bavarian vouchers with barcodes.

Identification	Sample ID	GenBank
Abraxas sylvata	BC ZSM Lep 24005	GU654900
Acasis appensata	BC ZSM Lep 24232	HM376826
Acasis viretata	BC ZSM Lep 01569	HQ601015
Acasis viretata	BC ZSM Lep 01568	HQ601016
Acasis viretata	BC ZSM Lep 02729	HQ601017
Acasis viretata	BC ZSM Lep 22849	GU686755
Aethalura punctulata	BC ZSM Lep 02779	HQ601018
Aethalura punctulata	BC ZSM Lep 21076	HQ601019
Aethalura punctulata	BC ZSM Lep 01316	HQ601020
Aethalura punctulata	BC ZSM Lep 22847	GU686753
Agriopis aurantiaria	BC ZSM Lep 02792	HQ601021
Agriopis aurantiaria	BC ZSM Lep 02791	HQ601022
Agriopis aurantiaria	BC ZSM Lep 21378	HQ601023
Agriopis aurantiaria	BC ZSM Lep 01225	HQ601024
Agriopis aurantiaria	BC ZSM Lep 01224	HQ601025
Agriopis aurantiaria	BC ZSM Lep 01189	HQ601026
Agriopis bajaria	BC ZSM Lep 24035	GU654875
Agriopis leucophaearia	BC ZSM Lep 11339	HQ601027
Agriopis leucophaearia	BC ZSM Lep 24033	GU654882
Agriopis leucophaearia	BC ZSM Lep 24034	HQ601028
Agriopis marginaria	BC ZSM Lep 01764	HQ601029
Agriopis marginaria	BC ZSM LepLa 0008	HQ601030
Agriopis marginaria	BC ZSM LepLa 0002	HQ601031
Agriopis marginaria	BC ZSM Lep 21142	HQ601032
Agriopis marginaria	BC ZSM Lep 01191	HQ601033
Agriopis marginaria	BC ZSM Lep 01190	HQ601034
Alcis bastelbergeri	BC ZSM Lep 24044	HQ601035
Alcis repandata	BC ZSM Lep 21065	HQ601036
Alcis repandata	BC ZSM Lep 21052	HQ601037
Alcis repandata	BC ZSM Lep 21379	HQ601038
Alcis repandata	BC ZSM Lep 21364	HQ601039
Alcis repandata	BC ZSM Lep 21340	HQ601040
Alcis repandata	BC ZSM Lep 01158	HQ601041
Alcis repandata	BC ZSM Lep 22065	GU686923
Alsophila aescularia	BC ZSM Lep 01165	HQ601042
Alsophila aescularia	BC ZSM Lep 01166	HQ601043
Alsophila aescularia	BC ZSM Lep 02766	HQ601044
Angerona prunaria	BC ZSM Lep 24991	GU654911
Angerona prunaria	BC ZSM Lep 01758	HQ601045
Angerona prunaria	BC ZSM Lep 21332	HQ601046
Angerona prunaria	BC ZSM Lep 01285	HQ601047

Anticlea badiata	BC ZSM Lep 01668	HQ601048
Anticlea badiata	BC ZSM Lep 01667	HQ601049
Anticlea badiata	BC ZSM Lep 01309	HQ601050
Anticlea badiata	BC ZSM Lep 02818	HQ601051
Anticlea badiata	BC ZSM Lep 02757	HQ601052
Anticlea badiata	BC ZSM Lep 23245	GU654938
Anticlea derivata	BC ZSM Lep 01682	HQ601053
Anticlea derivata	BC ZSM Lep 24129	GU687268
Anticlea derivata	BC ZSM Lep 24130	GU687269
Anticollix sparsata	BC ZSM Lep 21372	HQ601054
Anticollix sparsata	BC ZSM Lep 21051	HQ601055
Anticollix sparsata	BC ZSM Lep 01585	HQ601056
Anticollix sparsata	BC ZSM Lep 24218	GU687189
Apeira syringaria	BC ZSM Lep 01746	HQ601057
Apeira syringaria	BC ZSM Lep 21407	HQ601058
Apeira syringaria	BC ZSM Lep 22385	GU654862
Aplocera efformata	BC ZSM Lep 01561	HQ601059
Aplocera efformata	BC ZSM Lep 01182	HQ601060
Aplocera efformata	BC ZSM Lep 22368	GU686833
Aplocera plagiata	BC ZSM Lep 24220	GU687191
Aplocera praeformata	BC ZSM Lep 01215	HQ601061
Aplocera praeformata	BC ZSM Lep 24221	GU687184
Apocheima hispidaria	BC ZSM Lep 24029	GU654879
Archiearis parthenias	BC ZSM Lep 24003	HQ601062
Arichanna melanaria	BC ZSM Lep 21060	HQ601063
Arichanna melanaria	BC ZSM Lep 24045	GU654869
Aspitates gilvaria	BC ZSM Lep 01770	HQ601064
Aspitates gilvaria	BC ZSM Lep 24073	GU687318
Asthena albulata	BC ZSM Lep 21072	HQ601065
Asthena albulata	BC ZSM Lep 01288	HQ601066
Asthena anseraria	BC ZSM Lep 21102	HQ601067
Asthena anseraria	BC ZSM Lep 01578	HQ601068
Asthena anseraria	BC ZSM Lep 22358	GU686841
Baptria tibiale	BC ZSM Lep 24217	GU687188
Biston betularia	BC ZSM Lep 01163	HQ601069
Biston betularia	BC ZSM Lep 01162	HQ601070
Biston betularia	BC ZSM Lep 01071	HQ601071
Biston betularia	BC ZSM Lep 24032	GU654881
Biston betularia	BC ZSM Lep 15026	HQ601072
Biston betularia	BC ZSM Lep 28439	HQ601073
Biston strataria	BC ZSM Lep 02768	HQ601074
Biston strataria	BC ZSM Lep 02760	HQ601075
Biston strataria	BC ZSM Lep 24031	GU654880
Boudinotiana notha	BC ZSM Lep 24004	HM393529
Bupalus piniaria	BC ZSM Lep 21348	HQ601076
Bupalus piniaria	BC ZSM Lep 24051	GU687338
Cabera exanthemata	BC ZSM Lep 02802	HQ601077

Cabera exanthemata	BC ZSM Lep 01255	HQ601078
Cabera exanthemata	BC ZSM Lep 01246	HQ601079
Cabera exanthemata	BC ZSM Lep 01240	HQ601080
Cabera exanthemata	BC ZSM Lep 01144	HQ601081
Cabera exanthemata	BC ZSM Lep 01143	HQ601082
Cabera exanthemata	BC ZSM Lep 22435	GU654864
Cabera exanthemata	BC ZSM Lep 22068	HM903322
Cabera pusaria	BC ZSM Lep 02813	HQ601083
Cabera pusaria	BC ZSM Lep 21393	HQ601084
Cabera pusaria	BC ZSM Lep 01293	HQ601085
Cabera pusaria	BC ZSM Lep 01258	HQ601086
Campaea margaritaria	BC ZSM Lep 02781	HQ601087
Campaea margaritaria	BC ZSM Lep 01283	HQ601088
Campaea margaritaria	BC ZSM Lep 01177	HQ601089
Campaea margaritaria	BC ZSM Lep 01176	HQ601090
Camptogramma bilineata	BC ZSM Lep 02800	HQ601091
Camptogramma bilineata	BC ZSM Lep 21423	HQ601092
Camptogramma bilineata	BC ZSM Lep 01665	HQ601093
Camptogramma bilineata	BC ZSM Lep 01274	HQ601094
Camptogramma bilineata	BC ZSM Lep 01148	HQ601095
Camptogramma bilineata	BC ZSM Lep 01147	HQ601096
Carsia sororiata	BC ZSM Lep 24219	GU687190
Catarhoe cuculata	BC ZSM Lep 21389	HQ601097
Catarhoe cuculata	BC ZSM Lep 21368	HQ601098
Catarhoe cuculata	BC ZSM Lep 01276	HQ601099
Catarhoe rubidata	BC ZSM Lep 22335	GU686865
Catarhoe rubidata	BC ZSM Lep 24116	GU687279
Cepphis advenaria	BC ZSM Lep 24957	GU654910
Cepphis advenaria	BC ZSM Lep 01732	HQ601100
Cepphis advenaria	BC ZSM Lep 21073	HQ601101
Cepphis advenaria	BC ZSM Lep 24013	GU654893
Charissa ambiguata	BC ZSM Lep 01775	HQ601102
Charissa ambiguata	BC ZSM Lep 24058	GU687332
Charissa glaucinaria	BC ZSM Lep 28691	GU707269
Charissa glaucinaria	BC ZSM Lep 24060	GU687326
Charissa glaucinaria	BC ZSM Lep 24061	GU687327
Charissa obscurata	BC ZSM Lep 01774	HQ601103
Charissa obscurata	BC ZSM Lep 24057	GU687331
Charissa pullata	BC ZSM Lep 24059	GU687333
Chesias legatella	BC ZSM Lep 01559	HQ601104
Chiasmia clathrata	BC ZSM Lep 01724	HQ601105
Chiasmia clathrata	BC ZSM Lep 21413	HQ601106
Chiasmia clathrata	BC ZSM Lep 01305	HQ601107
Chlorissa cloraria	BC ZSM Lep 24077	GU687314
Chlorissa viridata	BC ZSM Lep 25447	HM391782
Chloroclysta siterata	BC ZSM Lep 01542	HQ601108
Chloroclysta siterata	BC ZSM Lep 02819	HQ601109

Chloroclysta siterata	BC ZSM Lep 02776	HQ601110
Chloroclysta siterata	BC ZSM Lep 02763	HQ601111
Chloroclysta siterata	BC ZSM LepLa 0012	HQ601112
Chloroclysta siterata	BC ZSM LepLa 0011	HQ601113
Chloroclysta siterata	BC ZSM Lep 01282	HQ601114
Chloroclysta siterata	BC ZSM Lep 01277	HQ601115
Chloroclysta siterata	BC ZSM Lep 01254	HQ601116
Chloroclysta siterata	BC ZSM Lep 01253	HQ601117
Chloroclysta siterata	BC ZSM Lep 01227	HQ601118
Chloroclysta siterata	BC ZSM Lep 23246	GU654939
Chloroclysta siterata	BC ZSM Lep 24147	GU687248
Chloroclystis v-ata	BC ZSM Lep 01556	HQ601119
Chloroclystis v-ata	BC ZSM Lep 12447	HQ601120
Chloroclystis v-ata	BC ZSM Lep 21375	HQ601121
Chloroclystis v-ata	BC ZSM Lep 01154	HQ601122
Chloroclystis v-ata	BC ZSM Lep 01153	HQ601123
Chloroclystis v-ata	BC ZSM Lep 22840	GU686761
Cidaria fulvata	BC ZSM Lep 21349	HQ601124
Cidaria fulvata	BC ZSM Lep 01138	HQ601125
Cidaria fulvata	BC ZSM Lep 24148	GU687249
Cleora cinctaria	BC ZSM Lep 24039	GU654872
Coenotephria salicataAH01By	BC ZSM Lep 01672	HQ601126
Coenotephria salicataAH01By	BC ZSM Lep 24139	GU687256
Coenotephria tophaceata	BC ZSM Lep 01679	HQ601127
Coenotephria tophaceata	BC ZSM Lep 24141	GU687258
Colostygia aptata	BC ZSM Lep 01688	HQ601128
Colostygia aptata	BC ZSM Lep 24167	GU687233
Colostygia aqueata	BC ZSM Lep 24170	GU687229
Colostygia kollariaria	BC ZSM Lep 24172	HM376821
Colostygia olivata	BC ZSM Lep 01690	HQ601129
Colostygia olivata	BC ZSM Lep 21400	HQ601130
Colostygia olivata	BC ZSM Lep 01203	HQ601131
Colostygia olivata	BC ZSM Lep 24168	GU687234
Colostygia olivata	BC ZSM Lep 24169	GU687235
Colostygia pectinataria	BC ZSM Lep 21356	HQ601132
Colostygia pectinataria	BC ZSM Lep 01303	HQ601133
Colostygia pectinataria	BC ZSM Lep 24173	GU687231
Colostygia turbata	BC ZSM Lep 01210	HQ601134
Colostygia turbata	BC ZSM Lep 24171	GU687230
Colotois pennaria	BC ZSM Lep 02785	HQ601135
Colotois pennaria	BC ZSM Lep 00071	HQ601136
Colotois pennaria	BC ZSM Lep 00070	HQ601137
Colotois pennaria	BC ZSM Lep 00069	HQ601138
Colotois pennaria	BC ZSM Lep 21362	HQ601139
Colotois pennaria	BC ZSM Lep 01223	HQ601140
Cosmorhoe ocellata	BC ZSM Lep 01259	HQ601141
Crocallis elinguaria	BC ZSM Lep 00081	HQ601142

Crocallis elinguaria	BC ZSM Lep 00080	HQ601143
Crocallis elinguaria	BC ZSM Lep 21414	HQ601144
Crocallis elinguaria	BC ZSM Lep 24028	GU654886
Cyclophora albipunctata	BC ZSM Lep 24080	GU687316
Cyclophora annularia	BC ZSM Lep 28416	GU707364
Cyclophora linearia	BC ZSM Lep 02774	HQ601145
Cyclophora linearia	BC ZSM Lep 24081	GU687310
Cyclophora punctaria	BC ZSM LepLa 0014	HQ601146
Cyclophora punctaria	BC ZSM LepLa 0013	HQ601147
Cyclophora punctaria	BC ZSM Lep 15023	HQ601148
Deileptenia ribeata	BC ZSM Lep 21056	HQ601149
Deileptenia ribeata	BC ZSM Lep 24041	GU654874
Deileptenia ribeata	BC ZSM Lep 24040	GU654873
Dysstroma citrata	BC ZSM Lep 01209	HQ601150
Dysstroma citrata	BC ZSM Lep 22025	GU686949
Dysstroma truncata	BC ZSM Lep 24974	GU654957
Dysstroma truncata	BC ZSM Lep 01273	HQ601151
Dysstroma truncata	BC ZSM Lep 02730	HQ601152
Dysstroma truncata	BC ZSM Lep 21355	HQ601153
Dysstroma truncata	BC ZSM Lep 37460	HQ563590
Ecliptopera capitata	BC ZSM Lep 21352	HQ601154
Ecliptopera capitata	BC ZSM Lep 21331	HQ601155
Ecliptopera capitata	BC ZSM Lep 24145	GU687254
Ecliptopera capitata	BC ZSM Lep 24146	GU687255
Ecliptopera silaceata	BC ZSM Lep 24925	GU654956
Ecliptopera silaceata	BC ZSM Lep 01534	HQ601156
Ecliptopera silaceata	BC ZSM Lep 21099	HQ601157
Ecliptopera silaceata	BC ZSM Lep 01297	HQ601158
Ecliptopera silaceata	BC ZSM Lep 01264	HQ601159
Ecliptopera silaceata	BC ZSM Lep 01186	HQ601160
Ectropis crepuscularia	BC ZSM Lep 21067	HQ601161
Ectropis crepuscularia	BC ZSM Lep 21053	HQ601162
Ectropis crepuscularia	BC ZSM LepLa 0003	HQ601163
Ectropis crepuscularia	BC ZSM Lep 06317	HQ601164
Ectropis crepuscularia	BC ZSM Lep 06316	HQ601165
Ectropis crepuscularia	BC ZSM Lep 06315	HQ601166
Ectropis crepuscularia	BC ZSM Lep 06314	HQ601167
Ectropis crepuscularia	BC ZSM Lep 06313	HQ601168
Ectropis crepuscularia	BC ZSM Lep 06312	HQ601169
Ectropis crepuscularia	BC ZSM Lep 06310	HQ601170
Ectropis crepuscularia	BC ZSM Lep 06309	HQ601171
Ectropis crepuscularia	BC ZSM Lep 21093	HQ601172
Ectropis crepuscularia	BC ZSM Lep 21089	HQ601173
Ectropis crepuscularia	BC ZSM Lep 21088	HQ601174
Ectropis crepuscularia	BC ZSM Lep 21086	HQ601175
Ectropis crepuscularia	BC ZSM Lep 01265	HQ601176
Ectropis crepuscularia	BC ZSM Lep 01194	HQ601177

Ectropis crepuscularia	BC ZSM Lep 01193	HQ601178
Ectropis crepuscularia	BC ZSM Lep 01099	HQ601179
Ectropis crepuscularia	BC ZSM Lep 01098	HQ601180
Ectropis crepuscularia	BC ZSM Lep 01096	HQ601181
Ectropis crepuscularia	BC ZSM Lep 01095	HQ601182
Ectropis crepuscularia	BC ZSM Lep 01094	HQ601183
Ectropis crepuscularia	BC ZSM Lep 01093	HQ601184
Ectropis crepuscularia	BC ZSM Lep 01092	HQ601185
Ectropis crepuscularia	BC ZSM Lep 23243	GU654903
Ectropis crepuscularia	BC ZSM Lep 25641	GU707179
Ectropis crepuscularia	BC ZSM Lep 22756	GU686832
Ectropis crepuscularia	BC ZSM Lep 21116	HQ601186
Electrophaes corylata	BC ZSM Lep 01523	HQ601187
Elophos caelibaria	BC ZSM Lep 24065	GU687325
Elophos dilucidaria	BC ZSM Lep 01782	HQ601188
Elophos dilucidaria	BC ZSM Lep 01781	HQ601189
Elophos dilucidaria	BC ZSM Lep 01780	HQ601190
Elophos dilucidaria	BC ZSM Lep 22066	GU686918
Elophos dilucidaria	BC ZSM Lep 24062	GU687328
Elophos dilucidaria	BC ZSM Lep 24063	GU687329
Elophos serotinaria	BC ZSM Lep 24067	HM376817
Elophos vittaria	BC ZSM Lep 24064	GU687324
Elophos zelleraria	BC ZSM Lep 01784	HQ601191
Elophos zelleraria	BC ZSM Lep 24066	HQ601192
Ematurga atomaria	BC ZSM Lep 24050	HQ601193
Ennomos alniaria	BC ZSM Lep 22351	GU686850
Ennomos autumnaria	BC ZSM Lep 24024	HQ601194
Ennomos erosaria	BC ZSM Lep 21058	HQ601195
Ennomos erosaria	BC ZSM Lep 21478	HQ601196
Ennomos erosaria	BC ZSM Lep 21476	HQ601197
Ennomos erosaria	BC ZSM Lep 24025	GU654883
Ennomos erosaria	BC ZSM Lep 27062	GU707117
Ennomos quercinaria	BC ZSM Lep 01747	HQ601198
Ennomos quercinaria	BC ZSM Lep 21061	HQ601199
Ennomos quercinaria	BC ZSM Lep 21415	HQ601200
Ennomos quercinaria	BC ZSM Lep 21406	HQ601201
Ennomos quercinaria	BC ZSM Lep 28437	GU707361
Entephria caesiata	BC ZSM Lep 01671	HQ601202
Entephria caesiata	BC ZSM Lep 22056	GU686924
Entephria caesiata	BC ZSM Lep 24127	GU687266
Entephria caesiata	BC ZSM Lep 24128	GU687267
Entephria cyanata	BC ZSM Lep 24124	GU687271
Entephria flavata	BC ZSM Lep 24123	GU687270
Entephria flavicinctata	BC ZSM Lep 24125	GU687272
Entephria infidaria	BC ZSM Lep 24126	GU687273
Entephria nobiliaria	BC ZSM Lep 01676	HQ601203
Entephria nobiliaria	BC ZSM Lep 24122	GU687277

Epilobophora sabinata	BC ZSM Lep 24228	HM376825
Epione repandaria	BC ZSM Lep 21360	HQ601204
Epione repandaria	BC ZSM Lep 01278	HQ601205
Epione repandaria	BC ZSM Lep 01174	HQ601206
Epione vespertaria	BC ZSM Lep 24019	HM376691
Epirrhoe alternata	BC ZSM Lep 24926	GU654955
Epirrhoe alternata	BC ZSM Lep 02811	HQ601207
Epirrhoe alternata	BC ZSM Lep 21401	HQ601208
Epirrhoe alternata	BC ZSM Lep 01684	HQ601209
Epirrhoe alternata	BC ZSM Lep 01666	HQ601210
Epirrhoe alternata	BC ZSM Lep 01188	HQ601211
Epirrhoe alternata	BC ZSM Lep 01187	HQ601212
Epirrhoe galiata	BC ZSM Lep 01664	HQ601213
Epirrhoe galiata	BC ZSM Lep 24120	GU687275
Epirrhoe galiata	BC ZSM Lep 24121	GU687276
Epirrhoe hastulata	BC ZSM Lep 01657	HQ601214
Epirrhoe molluginata	BC ZSM Lep 01661	HQ601215
Epirrhoe molluginata	BC ZSM Lep 24119	GU687274
Epirrhoe rivata	BC ZSM Lep 28418	HQ601216
Epirrhoe rivata	BC ZSM Lep 27652	GU688408
Epirrhoe tristata	BC ZSM Lep 01660	HQ601217
Epirrhoe tristata	BC ZSM Lep 01659	HQ601218
Epirrhoe tristata	BC ZSM Lep 24117	GU687280
Epirrhoe tristata	BC ZSM Lep 24118	GU687281
Epirrita autumnata	BC ZSM Lep 21472	HQ601219
Epirrita autumnata	BC ZSM Lep 01281	HQ601220
Epirrita autumnata	BC ZSM Lep 01157	HQ601221
Epirrita autumnata	BC ZSM Lep 24199	GU687202
Epirrita autumnata	BC ZSM Lep 22074	GU686916
Epirrita christyi	BC ZSM Lep 21471	HQ601222
Epirrita christyi	BC ZSM Lep 21124	HQ601223
Epirrita christyi	BC ZSM Lep 21123	HQ601224
Epirrita christyi	BC ZSM Lep 21119	HQ601225
Epirrita christyi	BC ZSM Lep 21096	HQ601226
Epirrita christyi	BC ZSM Lep 01251	HQ601227
Epirrita christyi	BC ZSM Lep 01250	HQ601228
Epirrita christyi	BC ZSM Lep 01168	HQ601229
Epirrita christyi	BC ZSM Lep 24198	GU687208
Epirrita christyi	BC ZSM Lep 28471	HM391891
Epirrita dilutata	BC ZSM Lep 02784	HQ601230
Epirrita dilutata	BC ZSM Lep 01248	HQ601231
Epirrita dilutata	BC ZSM Lep 01228	HQ601232
Erannis defoliaria	BC ZSM Lep 02794	HQ601233
Erannis defoliaria	BC ZSM Lep 02793	HQ601234
Erannis defoliaria	BC ZSM Lep 02790	HQ601235
Erannis defoliaria	BC ZSM Lep 21121	HQ601236
Erannis defoliaria	BC ZSM Lep 01271	HQ601237

Erannis defoliaria	BC ZSM Lep 01270	HQ601238
Erannis defoliaria	BC ZSM Lep 01269	HQ601239
Erannis defoliaria	BC ZSM Lep 01226	HQ601240
Euchoeca nebulata	BC ZSM Lep 24927	GU654954
Euchoeca nebulata	BC ZSM Lep 21391	HQ601241
Euchoeca nebulata	BC ZSM Lep 01235	HQ601242
Eulithis populata	BC ZSM Lep 01216	HQ601243
Eulithis populata	BC ZSM Lep 21068	HQ601244
Eulithis populata	BC ZSM Lep 22058	GU686925
Eulithis prunata	BC ZSM Lep 21376	HQ601245
Eulithis prunata	BC ZSM Lep 01243	HQ601246
Eulithis prunata	BC ZSM Lep 22411	GU654919
Eulithis prunata	BC ZSM Lep 01505	HQ601247
Euphyia adumbraria	BC ZSM Lep 24194	HM376822
Euphyia biangulata	BC ZSM Lep 24192	GU687211
Euphyia frustata	BC ZSM Lep 24195	GU687206
Euphyia scripturata	BC ZSM Lep 24196	GU687207
Euphyia unangulata	BC ZSM Lep 28421	GU707363
Euphyia unangulata	BC ZSM Lep 24193	GU687212
Eupithecia abbreviata	BC ZSM Lep 01610	HQ601248
Eupithecia abbreviata	BC ZSM Lep 01602	HQ601249
Eupithecia abbreviata	BC ZSM Lep 23239	GU654936
Eupithecia abbreviata	BC ZSM Lep 22828	GU686768
Eupithecia abietaria	BC ZSM Lep 24989	GU654953
Eupithecia abietaria	BC ZSM Lep 25610	GU707201
Eupithecia abietaria	BC ZSM Lep 22771	GU686818
Eupithecia absinthiata	BC ZSM Lep 21480	HQ601250
Eupithecia absinthiata	BC ZSM Lep 21474	HQ601251
Eupithecia absinthiata	BC ZSM Lep 01596	HQ601252
Eupithecia absinthiata	BC ZSM Lep 01586	HQ601253
Eupithecia absinthiata	BC ZSM Lep 22395	GU654926
Eupithecia absinthiata	BC ZSM Lep 22377	GU654931
Eupithecia absinthiata	BC ZSM Lep 22795	GU686794
Eupithecia absinthiata	BC ZSM Lep 22374	HM393648
Eupithecia absinthiata	BC ZSM Lep 22373	GU686831
Eupithecia absinthiata	BC ZSM Lep 22294	GU686902
Eupithecia absinthiata	BC ZSM Lep 22287	GU686899
Eupithecia actaeata	BC ZSM Lep 02746	HQ601254
Eupithecia actaeata	BC ZSM Lep 22786	GU686809
Eupithecia analoga	BC ZSM Lep 22772	GU686819
Eupithecia assimilata	BC ZSM Lep 24988	GU654949
Eupithecia assimilata	BC ZSM Lep 21475	HQ601255
Eupithecia assimilata	BC ZSM Lep 02741	HQ601256
Eupithecia assimilata	BC ZSM Lep 21105	HQ601257
Eupithecia assimilata	BC ZSM Lep 21104	HQ601258
Eupithecia assimilata	BC ZSM Lep 02770	HQ601259
Eupithecia assimilata	BC ZSM Lep 22798	GU686796

Eupithecia cauchiata	BC ZSM Lep 01594	HQ601260
Eupithecia cauchiata	BC ZSM Lep 22793	GU686800
Eupithecia cauchiata	BC ZSM Lep 22792	GU686799
Eupithecia centaureata	BC ZSM Lep 02749	HQ601261
Eupithecia centaureata	BC ZSM Lep 22784	GU686807
Eupithecia conterminata	BC ZSM Lep 23025	GU654940
Eupithecia conterminata	BC ZSM Lep 22837	HM393660
Eupithecia denotata	BC ZSM Lep 22322	HM393642
Eupithecia denotata	BC ZSM Lep 22802	HM376795
Eupithecia distinctaria	BC ZSM Lep 22819	HM393656
Eupithecia distinctaria	BC ZSM Lep 22818	GU686777
Eupithecia distinctaria	BC ZSM Lep 22783	GU686806
Eupithecia dodoneata	BC ZSM Lep 24947	GU654951
Eupithecia dodoneata	BC ZSM Lep 01612	HQ601262
Eupithecia dodoneata	BC ZSM Lep 22830	GU686770
Eupithecia dodoneata	BC ZSM Lep 22829	GU686769
Eupithecia egenaria	BC ZSM Lep 15018	HQ601263
Eupithecia egenaria	BC ZSM Lep 22781	GU686812
Eupithecia exiguata	BC ZSM Lep 01545	HQ601264
Eupithecia exiguata	BC ZSM Lep 02734	HQ601265
Eupithecia exiguata	BC ZSM Lep 21333	HQ601266
Eupithecia exiguata	BC ZSM Lep 21152	HQ601267
Eupithecia exiguata	BC ZSM Lep 21151	HQ601268
Eupithecia exiguata	BC ZSM Lep 21077	HQ601269
Eupithecia exiguata	BC ZSM Lep 22775	GU686814
Eupithecia exiguata	BC ZSM Lep 22774	GU686821
Eupithecia expallidata	BC ZSM Lep 01591	HQ601270
Eupithecia expallidata	BC ZSM Lep 22797	GU686795
Eupithecia extraversaria	BC ZSM Lep 22782	GU686813
Eupithecia goossensiata	BC ZSM Lep 01593	HQ601271
Eupithecia goossensiata	BC ZSM Lep 22796	HM393654
Eupithecia haworthiata	BC ZSM Lep 21370	HQ601272
Eupithecia haworthiata	BC ZSM Lep 22765	GU686828
Eupithecia haworthiata	BC ZSM Lep 22326	GU686875
Eupithecia icterata	BC ZSM Lep 21110	HQ601273
Eupithecia icterata	BC ZSM Lep 01597	HQ601274
Eupithecia icterata	BC ZSM Lep 22391	GU654932
Eupithecia icterata	BC ZSM Lep 28744	GU707400
Eupithecia icterata	BC ZSM Lep 22806	GU686788
Eupithecia immundata	BC ZSM Lep 01514	HQ601275
Eupithecia immundata	BC ZSM Lep 22766	GU686829
Eupithecia immundata	BC ZSM Lep 28432	HQ601276
Eupithecia impurata	BC ZSM Lep 22811	GU686785
Eupithecia impurata	BC ZSM Lep 22808	GU686790
Eupithecia indigata	BC ZSM Lep 02748	HQ601277
Eupithecia indigata	BC ZSM Lep 01609	HQ601278
Eupithecia indigata	BC ZSM Lep 22821	HM393657

Eupithecia indigata	BC ZSM Lep 28431	HQ601279
Eupithecia innotata	BC ZSM Lep 01601	HQ601280
Eupithecia innotata	BC ZSM Lep 01600	HQ601281
Eupithecia insigniata	BC ZSM Lep 22776	GU686815
Eupithecia intricata	BC ZSM Lep 24946	GU654952
Eupithecia intricata	BC ZSM Lep 21154	HQ601282
Eupithecia intricata	BC ZSM Lep 01295	HQ601283
Eupithecia intricata	BC ZSM Lep 01171	HQ601284
Eupithecia intricata	BC ZSM Lep 15025	HQ601285
Eupithecia intricata	BC ZSM Lep 22788	GU686803
Eupithecia intricata	BC ZSM Lep 22312	GU686880
Eupithecia inturbata	BC ZSM Lep 01511	HQ601286
Eupithecia inturbata	BC ZSM Lep 21050	HQ601287
Eupithecia inturbata	BC ZSM Lep 01266	HQ601288
Eupithecia inturbata	BC ZSM Lep 22432	GU654918
Eupithecia inturbata	BC ZSM Lep 22408	GU654921
Eupithecia inturbata	BC ZSM Lep 28292	GU707325
Eupithecia inturbata	BC ZSM Lep 22764	GU686827
Eupithecia inturbata	BC ZSM Lep 22344	GU686858
Eupithecia inturbata	BC ZSM Lep 22327	HM393644
Eupithecia lanceata	BC ZSM Lep 02759	HQ601289
Eupithecia lanceata	BC ZSM Lep 01616	HQ601290
Eupithecia lanceata	BC ZSM Lep 01603	HQ601291
Eupithecia lanceata	BC ZSM Lep 02777	HQ601292
Eupithecia lanceata	BC ZSM Lep 02762	HQ601293
Eupithecia lanceata	BC ZSM Lep 22833	GU686765
Eupithecia lanceata	BC ZSM Lep 28430	HQ601294
Eupithecia lariciata	BC ZSM Lep 24954	GU654950
Eupithecia lariciata	BC ZSM Lep 02750	HQ601295
Eupithecia lariciata	BC ZSM Lep 22834	GU686766
Eupithecia linariata	BC ZSM Lep 01537	HQ601296
Eupithecia linariata	BC ZSM Lep 22325	GU686874
Eupithecia nanata	BC ZSM Lep 22824	GU686773
Eupithecia nanata	BC ZSM Lep 22823	GU686772
Eupithecia nanata	BC ZSM Lep 22320	GU686879
Eupithecia orphnata	BC ZSM Lep 22812	GU686786
Eupithecia pimpinellata	BC ZSM Lep 01599	HQ601297
Eupithecia pimpinellata	BC ZSM Lep 22822	GU686778
Eupithecia plumbeolata	BC ZSM Lep 21054	HQ601298
Eupithecia plumbeolata	BC ZSM Lep 22770	GU686825
Eupithecia plumbeolata	BC ZSM Lep 22769	GU686824
Eupithecia plumbeolata	BC ZSM Lep 22768	GU686823
Eupithecia pulchellata	BC ZSM Lep 28426	GU707360
Eupithecia pusillata	BC ZSM Lep 22832	GU686764
Eupithecia pusillata	BC ZSM Lep 22831	GU686771
Eupithecia pyreneata	BC ZSM Lep 01533	HQ601299
Eupithecia satyrata	BC ZSM Lep 15019	HQ601300

Eupithecia satyrata	BC ZSM Lep 22794	GU686801
Eupithecia satyrata	BC ZSM Lep 22334	GU686864
Eupithecia selinata	BC ZSM Lep 21385	HQ601301
Eupithecia selinata	BC ZSM Lep 21354	HQ601302
Eupithecia selinata	BC ZSM Lep 22785	GU686808
Eupithecia selinata	BC ZSM Lep 28429	HQ601303
Eupithecia semigraphata	BC ZSM Lep 22817	GU686776
Eupithecia semigraphata	BC ZSM Lep 22816	GU686782
Eupithecia semigraphata	BC ZSM Lep 22810	GU686784
Eupithecia semigraphata	BC ZSM Lep 22809	GU686783
Eupithecia silenata	BC ZSM Lep 22778	GU686817
Eupithecia sinuosaria	BC ZSM Lep 22820	HM376796
Eupithecia subfuscata	BC ZSM Lep 21479	HQ601304
Eupithecia subfuscata	BC ZSM Lep 21396	HQ601305
Eupithecia subfuscata	BC ZSM Lep 21384	HQ601306
Eupithecia subfuscata	BC ZSM Lep 21373	HQ601307
Eupithecia subfuscata	BC ZSM Lep 21371	HQ601308
Eupithecia subfuscata	BC ZSM Lep 21359	HQ601309
Eupithecia subfuscata	BC ZSM Lep 21343	HQ601310
Eupithecia subfuscata	BC ZSM Lep 21338	HQ601311
Eupithecia subfuscata	BC ZSM Lep 22396	GU654929
Eupithecia subfuscata	BC ZSM Lep 22394	GU654928
Eupithecia subfuscata	BC ZSM Lep 22380	GU654934
Eupithecia subfuscata	BC ZSM Lep 22804	GU686793
Eupithecia subfuscata	BC ZSM Lep 22803	GU686792
Eupithecia subfuscata	BC ZSM Lep 22336	GU686866
Eupithecia subfuscata	BC ZSM Lep 22331	GU686870
Eupithecia subfuscata	BC ZSM Lep 22330	GU686869
Eupithecia subfuscata	BC ZSM Lep 22329	GU686868
Eupithecia subfuscata	BC ZSM Lep 22321	GU686872
Eupithecia subfuscata	BC ZSM Lep 22284	GU686904
Eupithecia subumbrata	BC ZSM Lep 22815	GU686781
Eupithecia subumbrata	BC ZSM Lep 22814	GU686780
Eupithecia subumbrata	BC ZSM Lep 22813	GU686779
Eupithecia succenturiata	BC ZSM Lep 22807	GU686789
Eupithecia tantillaria	BC ZSM Lep 02753	HQ601312
Eupithecia tantillaria	BC ZSM Lep 02747	HQ601313
Eupithecia tantillaria	BC ZSM Lep 21153	HQ601314
Eupithecia tantillaria	BC ZSM Lep 01611	HQ601315
Eupithecia tantillaria	BC ZSM Lep 01604	HQ601316
Eupithecia tantillaria	BC ZSM Lep 01170	HQ601317
Eupithecia tantillaria	BC ZSM Lep 15020	HQ601318
Eupithecia tantillaria	BC ZSM Lep 02769	HQ601319
Eupithecia tantillaria	BC ZSM Lep 22836	GU686767
Eupithecia tantillaria	BC ZSM Lep 22835	HM393659
Eupithecia tenuiata	BC ZSM Lep 22409	GU654922
Eupithecia tenuiata	BC ZSM Lep 22763	HM393653

Eupithecia tripunctaria	BC ZSM Lep 12448	HQ601320
Eupithecia tripunctaria	BC ZSM Lep 21420	HQ601321
Eupithecia tripunctaria	BC ZSM Lep 01268	HQ601322
Eupithecia tripunctaria	BC ZSM Lep 22388	HQ601323
Eupithecia tripunctaria	BC ZSM Lep 28291	GU707327
Eupithecia tripunctaria	BC ZSM Lep 22801	GU686791
Eupithecia tripunctaria	BC ZSM Lep 22800	HM393655
Eupithecia tripunctaria	BC ZSM Lep 22291	HM393639
Eupithecia tripunctaria	BC ZSM Lep 22073	HM393634
Eupithecia trisignaria	BC ZSM Lep 01517	HQ601324
Eupithecia trisignaria	BC ZSM Lep 22787	GU686802
Eupithecia valerianata	BC ZSM Lep 21374	HQ601325
Eupithecia valerianata	BC ZSM Lep 22777	GU686816
Eupithecia venosata	BC ZSM Lep 21388	HQ601326
Eupithecia venosata	BC ZSM Lep 22780	GU686811
Eupithecia venosata	BC ZSM Lep 22779	GU686810
Eupithecia veratraria	BC ZSM Lep 21059	HQ601327
Eupithecia veratraria	BC ZSM Lep 21055	HQ601328
Eupithecia veratraria	BC ZSM Lep 22072	HM393633
Eupithecia veratraria	BC ZSM Lep 22791	GU686798
Eupithecia veratraria	BC ZSM Lep 22790	GU686805
Eupithecia veratraria	BC ZSM Lep 22789	GU686804
Eupithecia virgaureata	BC ZSM Lep 22375	HM393649
Eupithecia virgaureata	BC ZSM Lep 01515	HQ601329
Eupithecia virgaureata	BC ZSM Lep 02751	HQ601330
Eupithecia virgaureata	BC ZSM Lep 02745	HQ601331
Eupithecia virgaureata	BC ZSM Lep 21113	HQ601332
Eupithecia virgaureata	BC ZSM Lep 22433	GU654914
Eupithecia virgaureata	BC ZSM Lep 22402	GU654925
Eupithecia virgaureata	BC ZSM Lep 22399	GU654924
Eupithecia virgaureata	BC ZSM Lep 22398	GU654923
Eupithecia virgaureata	BC ZSM Lep 22845	GU686758
Eupithecia virgaureata	BC ZSM Lep 22827	HM393658
Eupithecia virgaureata	BC ZSM Lep 22826	GU686775
Eupithecia vulgata	BC ZSM Lep 21078	HQ601333
Eupithecia vulgata	BC ZSM Lep 22406	GU654920
Eupithecia vulgata	BC ZSM Lep 02772	HQ601334
Eupithecia vulgata	BC ZSM Lep 22799	GU686797
Eupithecia vulgata	BC ZSM Lep 22324	HM393643
Eustroma reticulata	BC ZSM Lep 21115	HQ601335
Eustroma reticulata	BC ZSM Lep 28281	GU707334
Eustroma reticulata	BC ZSM Lep 24165	GU687239
Fagivorina arenaria	BC ZSM Lep 24048	GU654868
Gagitodes sagittata	BC ZSM Lep 24213	HQ601336
Gandaritis pyraliata	BC ZSM Lep 01507	HQ601337
Gandaritis pyraliata	BC ZSM Lep 21395	HQ601338
Gandaritis pyraliata	BC ZSM Lep 24144	GU687253

Geometra papilionaria	BC ZSM Lep 24075	HQ601339
Gnophos furvata	BC ZSM Lep 01771	HQ601340
Gnophos furvata	BC ZSM Lep 24054	GU687336
Gnophos obfuscata	BC ZSM Lep 22029	GU686952
Gnophos obfuscata	BC ZSM Lep 01773	HQ601341
Gnophos obfuscata	BC ZSM Lep 01772	HQ601342
Gnophos obfuscata	BC ZSM Lep 24055	GU687337
Gnophos obfuscata	BC ZSM Lep 24056	GU687330
Gymnoscelis rufifasciata	BC ZSM Lep 21381	HQ601343
Gymnoscelis rufifasciata	BC ZSM Lep 21342	HQ601344
Gymnoscelis rufifasciata	BC ZSM Lep 21108	HQ601345
Gymnoscelis rufifasciata	BC ZSM Lep 01605	HQ601346
Gymnoscelis rufifasciata	BC ZSM Lep 01169	HQ601347
Gymnoscelis rufifasciata	BC ZSM Lep 23238	GU654935
Gymnoscelis rufifasciata	BC ZSM Lep 22850	GU686756
Hemistola chrysoprasaria	BC ZSM Lep 21394	HQ601348
Hemistola chrysoprasaria	BC ZSM Lep 21062	HQ601349
Hemistola chrysoprasaria	BC ZSM Lep 22348	GU686855
Hemistola chrysoprasaria	BC ZSM Lep 22361	GU686844
Hemistola chrysoprasaria	BC ZSM Lep 24078	GU687315
Hemistola siciliana	BC ZSM Lep 06318	HQ601350
Hemithea aestivaria	BC ZSM Lep 24076	GU687319
Hemithea aestivaria	BC ZSM Lep 21366	HQ601351
Horisme aemulata	BC ZSM Lep 22069	GU686919
Horisme aemulata	BC ZSM Lep 24180	GU687223
Horisme radicaria	BC ZSM Lep 21097	HQ601352
Horisme radicaria	BC ZSM Lep 01275	HQ601353
Horisme radicaria	BC ZSM Lep 24179	GU687222
Horisme tersata	BC ZSM Lep 22319	GU686878
Horisme tersata	BC ZSM Lep 24178	GU687221
Horisme vitalbata	BC ZSM Lep 21049	HQ601354
Horisme vitalbata	BC ZSM Lep 22424	GU654912
Horisme vitalbata	BC ZSM Lep 24177	GU687228
Hydrelia flammeolaria	BC ZSM Lep 01136	HQ601355
Hydrelia flammeolaria	BC ZSM Lep 24224	GU687187
Hydrelia sylvata	BC ZSM Lep 24225	GU687181
Hydriomena furcata	BC ZSM Lep 21390	HQ601356
Hydriomena furcata	BC ZSM Lep 01696	HQ601357
Hydriomena furcata	BC ZSM Lep 24174	GU687225
Hydriomena impluviata	BC ZSM Lep 24973	GU654948
Hydriomena impluviata	BC ZSM Lep 02735	HQ601358
Hydriomena impluviata	BC ZSM Lep 21083	HQ601359
Hydriomena impluviata	BC ZSM Lep 01311	HQ601360
Hydriomena impluviata	BC ZSM Lep 24175	GU687226
Hydriomena ruberata	BC ZSM Lep 01700	HQ601361
Hydriomena ruberata	BC ZSM Lep 01699	HQ601362
Hydriomena ruberata	BC ZSM Lep 24176	GU687227

Hylaea fasciaria	BC ZSM Lep 21358	HQ601363
Hylaea fasciaria	BC ZSM Lep 21335	HQ601364
Hylaea fasciaria	BC ZSM Lep 21109	HQ601365
Hylaea fasciaria	BC ZSM Lep 21106	HQ601366
Hylaea fasciaria	BC ZSM Lep 01179	HQ601367
Hylaea fasciaria	BC ZSM Lep 28441	GU707359
Hylaea fasciaria	BC ZSM Lep 22282	GU686903
Hypomecis punctinalis	BC ZSM Lep 02780	HQ601368
Hypomecis punctinalis	BC ZSM Lep 02743	HQ601369
Hypomecis punctinalis	BC ZSM Lep 01289	HQ601370
Hypomecis punctinalis	BC ZSM Lep 24047	GU654871
Hypomecis roboraria	BC ZSM Lep 21363	HQ601371
Hypomecis roboraria	BC ZSM Lep 24046	GU654870
Hypoxystis pluviaria	BC ZSM Lep 24022	GU654889
Hypoxystis pluviaria	BC ZSM Lep 22357	GU686848
Hypoxystis pluviaria	BC ZSM Lep 22356	GU686847
Idaea aureolaria	BC ZSM Lep 24092	GU687305
Idaea aversata	BC ZSM Lep 21344	HQ601372
Idaea aversata	BC ZSM Lep 01145	HQ601373
Idaea biselata	BC ZSM Lep 21412	HQ601374
Idaea biselata	BC ZSM Lep 21404	HQ601375
Idaea biselata	BC ZSM Lep 01256	HQ601376
Idaea dilutaria	BC ZSM Lep 28279	GU707336
Idaea dimidiata	BC ZSM Lep 21411	HQ601377
Idaea dimidiata	BC ZSM Lep 21392	HQ601378
Idaea dimidiata	BC ZSM Lep 01241	HQ601379
Idaea emarginata	BC ZSM Lep 22293	GU686901
Idaea fuscovenosa	BC ZSM Lep 21397	HQ601380
Idaea fuscovenosa	BC ZSM Lep 01195	HQ601381
Idaea fuscovenosa	BC ZSM Lep 22431	HM393510
Idaea humiliata	BC ZSM Lep 28413	GU707366
Idaea humiliata	BC ZSM Lep 24098	GU687294
Idaea inquinata	BC ZSM Lep 01198	HQ601382
Idaea inquinata	BC ZSM Lep 01196	HQ601383
Idaea inquinata	BC ZSM Lep 25611	GU707202
Idaea muricata	BC ZSM Lep 22364	GU686838
Idaea seriata	BC ZSM Lep 02737	HQ601384
Idaea seriata	BC ZSM Lep 21334	HQ601385
Idaea seriata	BC ZSM Lep 01280	HQ601386
Idaea seriata	BC ZSM Lep 25612	GU707199
Idaea seriata	BC ZSM Lep 28414	GU707367
Idaea serpentata	BC ZSM Lep 24093	GU687298
Idaea serpentata	BC ZSM Lep 24094	GU687299
Idaea straminata	BC ZSM Lep 22290	GU686900
Idaea straminata	BC ZSM Lep 24100	GU687295
Idaea straminata	BC ZSM Lep 24101	GU687296
Idaea sylvestraria	BC ZSM Lep 22407	HM393502

Idaea sylvestraria	BC ZSM Lep 22365	HM393647
Idaea sylvestraria	BC ZSM Lep 22283	HM393637
Idaea sylvestraria	BC ZSM Lep 22292	HM393640
Idaea sylvestraria	BC ZSM Lep 24096	GU687300
Isturgia roraria	BC ZSM Lep 24010	GU654898
Jodis lactearia	BC ZSM Lep 02738	HQ601387
Jodis lactearia	BC ZSM Lep 21079	HQ601388
Jodis lactearia	BC ZSM Lep 22310	GU686886
Jodis putata	BC ZSM Lep 24079	HM376818
Jodis putata	BC ZSM Lep 28435	HQ601389
Lampropteryx otregiata	BC ZSM Lep 24972	GU654947
Lampropteryx otregiata	BC ZSM Lep 24138	GU687262
Lampropteryx otregiata	BC ZSM Lep 37470	HQ563600
Lampropteryx suffumata	BC ZSM Lep 01313	FJ376641
Lampropteryx suffumata	BC ZSM Lep 24137	GU687261
Lampropteryx suffumata	BC ZSM Lep 21090	HQ601390
Lampropteryx suffumata	BC ZSM Lep 01670	FJ376640
Ligdia adustata	BC ZSM Lep 02814	HQ601391
Ligdia adustata	BC ZSM Lep 02783	HQ601392
Ligdia adustata	BC ZSM Lep 01237	HQ601393
Ligdia adustata	BC ZSM Lep 01236	HQ601394
Ligdia adustata	BC ZSM Lep 01720	HQ601395
Lobophora halterata	BC ZSM Lep 01567	HQ601396
Lobophora halterata	BC ZSM Lep 01566	HQ601397
Lobophora halterata	BC ZSM Lep 01308	HQ601398
Lobophora halterata	BC ZSM Lep 22429	GU654917
Lobophora halterata	BC ZSM Lep 22393	GU654927
Lobophora halterata	BC ZSM Lep 23244	GU654937
Lobophora halterata	BC ZSM Lep 22848	GU686754
Lobophora halterata	BC ZSM Lep 24227	GU687183
Lomaspilis marginata	BC ZSM Lep 01685	HQ601399
Lomographa bimaculata	BC ZSM Lep 24928	GU654909
Lomographa bimaculata	BC ZSM Lep 02728	HQ601400
Lomographa bimaculata	BC ZSM Lep 21351	HQ601401
Lomographa bimaculata	BC ZSM Lep 22352	GU686851
Lomographa temerata	BC ZSM Lep 01294	HQ601402
Lycia hirtaria	BC ZSM Lep 02755	HQ601403
Lycia hirtaria	BC ZSM Lep 02754	HQ601404
Lycia hirtaria	BC ZSM Lep 11517	HQ601405
Lycia hirtaria	BC ZSM Lep 11516	HQ601406
Lycia hirtaria	BC ZSM Lep 11515	HQ601407
Lycia hirtaria	BC ZSM Lep 11514	HQ601408
Lycia hirtaria	BC ZSM Lep 11513	HQ601409
Lycia hirtaria	BC ZSM Lep 21087	HQ601410
Lycia hirtaria	BC ZSM Lep 22423	GU654865
Macaria alternata	BC ZSM Lep 21336	HQ601411
Macaria alternata	BC ZSM Lep 24007	GU654902

Macaria artesiaria	BC ZSM Lep 22289	HM393638
Macaria brunneata	BC ZSM Lep 21070	HQ601412
Macaria brunneata	BC ZSM Lep 22339	GU686861
Macaria brunneata	BC ZSM Lep 24011	GU654899
Macaria brunneata	BC ZSM Lep 22342	GU686856
Macaria fusca	BC ZSM Lep 24012	GU654892
Macaria liturata	BC ZSM Lep 21386	HQ601413
Macaria liturata	BC ZSM Lep 01306	HQ601414
Macaria liturata	BC ZSM Lep 01142	HQ601415
Macaria liturata	BC ZSM Lep 01141	HQ601416
Macaria liturata	BC ZSM Lep 24009	GU654897
Macaria notata	BC ZSM Lep 21416	HQ601417
Macaria notata	BC ZSM Lep 01722	HQ601418
Macaria notata	BC ZSM Lep 01180	HQ601419
Macaria notata	BC ZSM Lep 22363	HM393646
Macaria notata	BC ZSM Lep 22085	GU686912
Macaria notata	BC ZSM Lep 01721	HQ601420
Macaria notata	BC ZSM Lep 22414	GU654866
Macaria signaria	BC ZSM Lep 21405	HQ601421
Macaria signaria	BC ZSM Lep 01291	HQ601422
Macaria signaria	BC ZSM Lep 22064	GU686922
Macaria signaria	BC ZSM Lep 24008	GU654896
Macaria wauaria	BC ZSM Lep 21346	HQ601423
Macaria wauaria	BC ZSM Lep 01146	HQ601424
Macaria wauaria	BC ZSM Lep 22314	GU686882
Macaria wauaria	BC ZSM Lep 25616	GU707197
Martania taeniata	BC ZSM Lep 24200	GU687203
Melanthia alaudaria	BC ZSM Lep 24181	GU687224
Melanthia procellata	BC ZSM Lep 02810	HQ601425
Melanthia procellata	BC ZSM Lep 02809	HQ601426
Melanthia procellata	BC ZSM Lep 02797	HQ601427
Melanthia procellata	BC ZSM Lep 02731	HQ601428
Melanthia procellata	BC ZSM Lep 21367	HQ601429
Melanthia procellata	BC ZSM Lep 01233	HQ601430
Melanthia procellata	BC ZSM Lep 01184	HQ601431
Mesoleuca albicillata	BC ZSM Lep 24959	GU654946
Mesoleuca albicillata	BC ZSM Lep 01284	HQ601432
Mesoleuca albicillata	BC ZSM Lep 24131	GU687263
Mesotype didymata	BC ZSM Lep 24210	GU687198
Mesotype didymata	BC ZSM Lep 24211	GU687192
Mesotype parallelolineata	BC ZSM Lep 01520	HQ601433
Mesotype verberata	BC ZSM Lep 01205	HQ601434
Mesotype verberata	BC ZSM Lep 22060	HM393631
Mesotype verberata	BC ZSM Lep 22030	GU686946
Mesotype verberata	BC ZSM Lep 24215	GU687194
Minoa murinata	BC ZSM Lep 01583	HQ601435
Minoa murinata	BC ZSM Lep 24226	GU687182

Nebula nebulata	BC ZSM Lep 22057	HM393630
Nebula nebulata	BC ZSM Lep 24142	GU687259
Nebula nebulata	BC ZSM Lep 24143	GU687252
Nothocasis sertata	BC ZSM Lep 01570	HQ601436
Nothocasis sertata	BC ZSM Lep 01221	HQ601437
Nothocasis sertata	BC ZSM Lep 01571	HQ601438
Nothocasis sertata	BC ZSM Lep 22077	GU686917
Nothocasis sertata	BC ZSM Lep 24231	HM903324
Nothocasis sertata	BC ZSM Lep 37461	HQ563591
Odezia atrata	BC ZSM Lep 01580	HQ601439
Odezia atrata	BC ZSM Lep 01579	HQ601440
Odezia atrata	BC ZSM Lep 24222	GU687185
Odontopera bidentata	BC ZSM Lep 02742	HQ601441
Odontopera bidentata	BC ZSM Lep 01300	HQ601442
Odontopera bidentata	BC ZSM Lep 01299	HQ601443
Operophtera brumata	BC ZSM Lep 02795	HQ601444
Operophtera brumata	BC ZSM Lep 02789	HQ601445
Operophtera brumata	BC ZSM Lep 02788	HQ601446
Operophtera brumata	BC ZSM Lep 02786	HQ601447
Operophtera brumata	BC ZSM LepLa 0006	HQ601448
Operophtera brumata	BC ZSM LepLa 0005	HQ601449
Operophtera brumata	BC ZSM LepLa 0004	HQ601450
Operophtera brumata	BC ZSM LepLa 0001	HQ601451
Operophtera brumata	BC ZSM Lep 01229	HQ601452
Operophtera brumata	BC ZSM Lep 01202	HQ601453
Operophtera fagata	BC ZSM Lep 21473	HQ601454
Operophtera fagata	BC ZSM Lep 01167	HQ601455
Opisthograptis luteolata	BC ZSM Lep 02727	HQ601456
Opisthograptis luteolata	BC ZSM Lep 21081	HQ601457
Opisthograptis luteolata	BC ZSM Lep 24018	GU654891
Orthonama obstipata	BC ZSM Lep 21126	HQ601458
Orthonama obstipata	BC ZSM Lep 21118	HQ601459
Orthonama obstipata	BC ZSM Lep 01173	HQ601460
Orthonama obstipata	BC ZSM Lep 22389	GU654930
Orthonama vittata	BC ZSM Lep 24108	GU687287
Orthonama vittata	BC ZSM Lep 24109	GU687288
Ourapteryx sambucaria	BC ZSM Lep 01140	HQ601461
Ourapteryx sambucaria	BC ZSM Lep 01139	HQ601462
Pachycnemia hippocastanaria	BC ZSM Lep 01736	HQ601463
Pachycnemia hippocastanaria	BC ZSM Lep 24017	GU654890
Paradarisa consonaria	BC ZSM Lep 02744	HQ601464
Paradarisa consonaria	BC ZSM Lep 02740	HQ601465
Parectropis similaria	BC ZSM Lep 21084	HQ601466
Parectropis similaria	BC ZSM Lep 15017	HQ601467
Pareulype berberata	BC ZSM Lep 02805	HQ601468
Pareulype berberata	BC ZSM Lep 01267	HQ601469
Pareulype berberata	BC ZSM Lep 01244	HQ601470

Pareulype berberata	BC ZSM Lep 01152	HQ601471
Pasiphila chloerata	BC ZSM Lep 01557	HQ601472
Pasiphila chloerata	BC ZSM Lep 22842	GU686763
Pasiphila debiliata	BC ZSM Lep 22843	HM376797
Pasiphila rectangulata	BC ZSM Lep 27044	GU707135
Pasiphila rectangulata	BC ZSM Lep 21345	HQ601473
Pasiphila rectangulata	BC ZSM Lep 21339	HQ601474
Pasiphila rectangulata	BC ZSM Lep 01181	HQ601475
Pasiphila rectangulata	BC ZSM Lep 01156	HQ601476
Pasiphila rectangulata	BC ZSM Lep 01155	HQ601477
Pasiphila rectangulata	BC ZSM Lep 22841	GU686762
Pasiphila rectangulata	BC ZSM Lep 22337	GU686867
Pasiphila rectangulata	BC ZSM Lep 22333	GU686871
Pasiphila rectangulata	BC ZSM Lep 22286	GU686906
Pennithera firmata	BC ZSM Lep 21122	HQ601478
Pennithera firmata	BC ZSM Lep 21112	HQ601479
Pennithera firmata	BC ZSM Lep 01150	HQ601480
Pennithera firmata	BC ZSM Lep 01149	HQ601481
Pennithera firmata	BC ZSM Lep 24152	GU687244
Pennithera firmata	BC ZSM Lep 37463	HQ563593
Peribatodes rhomboidaria	BC ZSM Lep 01245	HQ601482
Peribatodes rhomboidaria	BC ZSM Lep 01231	HQ601483
Peribatodes rhomboidaria	BC ZSM Lep 01192	HQ601484
Peribatodes rhomboidaria	BC ZSM Lep 01161	HQ601485
Peribatodes rhomboidaria	BC ZSM Lep 01160	HQ601486
Peribatodes rhomboidaria	BC ZSM Lep 24038	GU654878
Peribatodes rhomboidaria	BC ZSM Lep 02808	HQ601487
Peribatodes rhomboidaria	BC ZSM Lep 02807	HQ601488
Peribatodes rhomboidaria	BC ZSM Lep 02806	HQ601489
Peribatodes secundaria	BC ZSM Lep 01767	HQ601490
Peribatodes secundaria	BC ZSM Lep 01766	HQ601491
Peribatodes secundaria	BC ZSM Lep 21409	HQ601492
Peribatodes secundaria	BC ZSM Lep 01208	HQ601493
Perizoma affinitata	BC ZSM Lep 01524	HQ601494
Perizoma affinitata	BC ZSM Lep 24201	GU687204
Perizoma albulata	BC ZSM Lep 24207	GU687195
Perizoma albulata	BC ZSM Lep 24208	GU687196
Perizoma alchemillata	BC ZSM Lep 02799	HQ601495
Perizoma alchemillata	BC ZSM Lep 21421	HQ601496
Perizoma alchemillata	BC ZSM Lep 21402	HQ601497
Perizoma alchemillata	BC ZSM Lep 21382	HQ601498
Perizoma alchemillata	BC ZSM Lep 21114	HQ601499
Perizoma alchemillata	BC ZSM Lep 21107	HQ601500
Perizoma alchemillata	BC ZSM Lep 01185	HQ601501
Perizoma alchemillata	BC ZSM Lep 24202	GU687205
Perizoma alchemillata	BC ZSM Lep 24204	GU687200
Perizoma alchemillata	BC ZSM Lep 22328	HM393645

Perizoma alchemillata	BC ZSM Lep 02812	HQ601502
Perizoma bifaciata	BC ZSM Lep 01510	HQ601503
Perizoma bifaciata	BC ZSM Lep 01509	HQ601504
Perizoma bifaciata	BC ZSM Lep 22378	GU654913
Perizoma blandiata	BC ZSM Lep 01548	HQ601505
Perizoma blandiata	BC ZSM Lep 24206	GU687201
Perizoma flavofasciata	BC ZSM Lep 24945	GU654945
Perizoma flavofasciata	BC ZSM Lep 01540	HQ601506
Perizoma flavofasciata	BC ZSM Lep 01529	HQ601507
Perizoma flavofasciata	BC ZSM Lep 24209	GU687197
Perizoma incultaria	BC ZSM Lep 24214	HM376824
Perizoma minorata	BC ZSM Lep 24205	HM376823
Perizoma obsoletata	BC ZSM Lep 01547	HQ601508
Perizoma obsoletata	BC ZSM Lep 24212	GU687193
Petrophora chlorosata	BC ZSM Lep 24014	GU654894
Petrophora chlorosata	BC ZSM Lep 22382	GU654867
Petrophora chlorosata	BC ZSM Lep 25617	GU707198
Phibalapteryx virgata	BC ZSM Lep 01619	HQ601509
Phibalapteryx virgata	BC ZSM Lep 24103	GU687290
Phigalia pilosaria	BC ZSM Lep 21141	HQ601510
Phigalia pilosaria	BC ZSM Lep 24030	HQ601511
Philereme transversata	BC ZSM Lep 21387	HQ601512
Philereme transversata	BC ZSM Lep 01713	HQ601513
Philereme transversata	BC ZSM Lep 25640	GU707182
Philereme transversata	BC ZSM Lep 24190	GU687209
Philereme transversata	BC ZSM Lep 24191	GU687210
Philereme vetulata	BC ZSM Lep 21377	HQ601514
Philereme vetulata	BC ZSM Lep 01712	HQ601515
Philereme vetulata	BC ZSM Lep 01711	HQ601516
Philereme vetulata	BC ZSM Lep 24189	GU687216
Plagodis dolabraria	BC ZSM Lep 24929	GU654908
Plagodis dolabraria	BC ZSM Lep 01734	HQ601517
Plagodis dolabraria	BC ZSM Lep 02782	HQ601518
Plagodis dolabraria	BC ZSM Lep 21082	HQ601519
Plagodis dolabraria	BC ZSM Lep 22362	GU686837
Plagodis dolabraria	BC ZSM Lep 24016	HQ601520
Plagodis pulveraria	BC ZSM Lep 24015	GU654895
Plemyria rubiginata	BC ZSM Lep 21337	HQ601521
Plemyria rubiginata	BC ZSM Lep 24150	GU687250
Plemyria rubiginata	BC ZSM Lep 24151	GU687251
Pseudopanthera maculata	BC ZSM Lep 24021	GU654888
Pseudopanthera maculata	BC ZSM Lep 24020	GU654887
Psodos alpinata	BC ZSM Lep 24072	GU687317
Psodos canaliculata	BC ZSM Lep 24069	GU687321
Psodos coracina	BC ZSM Lep 24071	GU687323
Psodos noricana	BC ZSM Lep 24070	GU687322
Psodos quadrifaria	BC ZSM Lep 24068	GU687320

Pterapherapteryx sexalata	BC ZSM Lep 24230	GU687179
Rheumaptera cervinalis	BC ZSM Lep 02765	HQ601522
Rheumaptera cervinalis	BC ZSM Lep 01302	HQ601523
Rheumaptera cervinalis	BC ZSM Lep 01301	HQ601524
Rheumaptera cervinalis	BC ZSM Lep 01298	HQ601525
Rheumaptera cervinalis	BC ZSM Lep 01129	HQ601526
Rheumaptera cervinalis	BC ZSM Lep 01595	HQ601527
Rheumaptera cervinalis	BC ZSM Lep 24184	GU687219
Rheumaptera hastata	BC ZSM Lep 24182	GU687217
Rheumaptera hastata	BC ZSM Lep 24183	GU687218
Rheumaptera undulata	BC ZSM Lep 21418	HQ601528
Rheumaptera undulata	BC ZSM Lep 21417	HQ601529
Rheumaptera undulata	BC ZSM Lep 24185	GU687220
Rheumaptera undulata	BC ZSM Lep 28417	HQ601530
Rhodostrophia vibicaria	BC ZSM Lep 24102	GU687297
Sciadia tenebraria	BC ZSM Lep 16890	FJ581453
Sciadia tenebraria	BC ZSM Lep 24037	GU654877
Sciadia tenebraria	BC ZSM Lep 24036	GU654876
Scopula floslactata	BC ZSM Lep 24958	HM391755
Scopula floslactata	BC ZSM Lep 02820	HQ601531
Scopula floslactata	BC ZSM Lep 21098	HQ601532
Scopula floslactata	BC ZSM Lep 28412	GU707368
Scopula immorata	BC ZSM Lep 01310	HQ601533
Scopula immorata	BC ZSM Lep 22338	GU686860
Scopula immorata	BC ZSM Lep 24082	GU687311
Scopula immorata	BC ZSM Lep 24083	GU687312
Scopula immutata	BC ZSM Lep 24089	GU687302
Scopula immutata	BC ZSM Lep 37471	HQ563601
Scopula incanata	BC ZSM Lep 24088	GU687309
Scopula marginepunctata	BC ZSM Lep 28411	HM902079
Scopula marginepunctata	BC ZSM Lep 24087	GU687308
Scopula nigropunctata	BC ZSM Lep 21347	HQ601534
Scopula nigropunctata	BC ZSM Lep 01315	HQ601535
Scopula nigropunctata	BC ZSM Lep 25615	GU707200
Scopula nigropunctata	BC ZSM Lep 24085	GU687306
Scopula ornata	BC ZSM Lep 21424	HQ601536
Scopula ornata	BC ZSM Lep 21080	HQ601537
Scopula ornata	BC ZSM Lep 22401	HM393499
Scopula ornata	BC ZSM Lep 22084	GU686911
Scopula ornata	BC ZSM Lep 24086	GU687307
Scopula rubiginata	BC ZSM Lep 21422	HQ601538
Scopula rubiginata	BC ZSM Lep 21419	HQ601539
Scopula rubiginata	BC ZSM Lep 22404	HM393500
Scopula subpunctaria	BC ZSM Lep 24091	GU687304
Scopula ternata	BC ZSM Lep 24090	GU687303
Scopula umbelaria	BC ZSM Lep 24084	GU687313
Scotopteryx bipunctaria	BC ZSM Lep 24105	GU687292

Scotopteryx bipunctaria	BC ZSM Lep 24106	GU687293
Scotopteryx chenopodiata	BC ZSM Lep 21111	HQ601540
Scotopteryx chenopodiata	BC ZSM Lep 01199	HQ601541
Scotopteryx chenopodiata	BC ZSM Lep 24107	GU687286
Scotopteryx luridata	BC ZSM Lep 25445	GU654944
Scotopteryx luridata	BC ZSM Lep 25768	GU707070
Scotopteryx moeniata	BC ZSM Lep 01632	HQ601542
Scotopteryx moeniata	BC ZSM Lep 24104	GU687291
Selenia dentaria	BC ZSM Lep 01307	HQ601543
Selenia dentaria	BC ZSM Lep 01257	HQ601544
Selenia dentaria	BC ZSM Lep 24026	GU654884
Selenia lunularia	BC ZSM Lep 24930	GU654907
Selenia lunularia	BC ZSM Lep 24027	GU654885
Selenia tetralunaria	BC ZSM Lep 01753	HQ601545
Selenia tetralunaria	BC ZSM Lep 02756	HQ601546
Selenia tetralunaria	BC ZSM Lep 01312	HQ601547
Siona lineata	BC ZSM Lep 24968	GU654906
Siona lineata	BC ZSM Lep 01768	HQ601548
Siona lineata	BC ZSM Lep 22369	GU686834
Siona lineata	BC ZSM Lep 22413	GU654863
Spargania luctuata	BC ZSM Lep 21477	HQ601549
Spargania luctuata	BC ZSM Lep 28420	GU707362
Stegania trimaculata	BC ZSM Lep 24006	GU654901
Thalera fimbrialis	BC ZSM Lep 21425	HQ601550
Thera britannica	BC ZSM Lep 24923	GU654943
Thera britannica	BC ZSM Lep 22070	HM393632
Thera cognata	BC ZSM Lep 22027	GU686950
Thera cognata	BC ZSM Lep 24163	GU687237
Thera juniperata	BC ZSM Lep 02787	HQ601551
Thera juniperata	BC ZSM Lep 01230	HQ601552
Thera juniperata	BC ZSM Lep 28419	GU707365
Thera juniperata	BC ZSM Lep 24164	GU687238
Thera obeliscata	BC ZSM Lep 01508	HQ601553
Thera obeliscata	BC ZSM Lep 02771	HQ601554
Thera obeliscata	BC ZSM Lep 02752	HQ601555
Thera obeliscata	BC ZSM Lep 02739	HQ601556
Thera obeliscata	BC ZSM Lep 21357	HQ601557
Thera obeliscata	BC ZSM Lep 21120	HQ601558
Thera obeliscata	BC ZSM Lep 21057	HQ601559
Thera obeliscata	BC ZSM Lep 21046	HQ601560
Thera obeliscata	BC ZSM Lep 01134	HQ601561
Thera obeliscata	BC ZSM Lep 01131	HQ601562
Thera obeliscata	BC ZSM Lep 01130	HQ601563
Thera obeliscata	BC ZSM Lep 15024	HQ601564
Thera obeliscata	BC ZSM Lep 15021	HQ601565
Thera obeliscata	BC ZSM Lep 15016	HQ601566
Thera obeliscata	BC ZSM Lep 15015	HQ601567

Thera obeliscata	BC ZSM Lep 15014	HQ601568
Thera obeliscata	BC ZSM Lep 22434	GU654915
Thera obeliscata	BC ZSM Lep 22075	HM393635
Thera obeliscata	BC ZSM Lep 22062	GU686920
Thera obeliscata	BC ZSM Lep 24155	GU687247
Thera obeliscata	BC ZSM Lep 24156	GU687240
Thera obeliscata	BC ZSM Lep 24157	GU687241
Thera obeliscata	BC ZSM Lep 37467	HQ563597
Thera variata	BC ZSM Lep 01553	HQ601569
Thera variata	BC ZSM Lep 21470	HQ601570
Thera variata	BC ZSM Lep 21350	HQ601571
Thera variata	BC ZSM Lep 01132	HQ601572
Thera variata	BC ZSM Lep 15022	HQ601573
Thera variata	BC ZSM Lep 22392	GU654933
Thera variata	BC ZSM Lep 22076	HM393636
Thera variata	BC ZSM Lep 22061	GU686926
Thera variata	BC ZSM Lep 24153	GU687245
Thera variata	BC ZSM Lep 24154	GU687246
Thera variata	BC ZSM Lep 37473	HQ563603
Thera vetustata	BC ZSM Lep 24162	GU687236
Theria primaria	BC ZSM Lep 25428	GU654905
Theria primaria	BC ZSM Lep 24053	GU687335
Theria rupicapraria	BC ZSM Lep 23191	GU654904
Theria rupicapraria	BC ZSM Lep 24052	GU687334
Timandra comae	BC ZSM Lep 24922	HM391754
Timandra comae	BC ZSM Lep 21410	HQ601574
Timandra comae	BC ZSM Lep 21341	HQ601575
Timandra comae	BC ZSM Lep 01279	HQ601576
Timandra comae	BC ZSM Lep 01272	HQ601577
Timandra comae	BC ZSM Lep 01175	HQ601578
Timandra comae	BC ZSM Lep 37459	HQ563589
Trichopteryx carpinata	BC ZSM Lep 02775	HQ601579
Trichopteryx carpinata	BC ZSM Lep 02764	HQ601580
Trichopteryx polycommata	BC ZSM Lep 02767	HQ601581
Trichopteryx polycommata	BC ZSM Lep 21091	HQ601582
Trichopteryx polycommata	BC ZSM Lep 01164	HQ601583
Trichopteryx polycommata	BC ZSM Lep 01572	HQ601584
Trichopteryx polycommata	BC ZSM Lep 24229	GU687178
Triphosa dubitata	BC ZSM Lep 02761	HQ601585
Triphosa dubitata	BC ZSM Lep 01708	HQ601586
Triphosa dubitata	BC ZSM Lep 24188	GU687215
Triphosa sabaudiata	BC ZSM Lep 24186	GU687213
Triphosa sabaudiata	BC ZSM Lep 24187	GU687214
Venusia blomeri	BC ZSM Lep 21103	HQ601587
Venusia blomeri	BC ZSM Lep 21048	HQ601588
Venusia blomeri	BC ZSM Lep 01575	HQ601589
Venusia cambrica	BC ZSM Lep 24223	GU687186

Xanthorhoe biriviata	BC ZSM Lep 24951	GU654941
Xanthorhoe biriviata	BC ZSM Lep 21353	HQ601590
Xanthorhoe biriviata	BC ZSM Lep 24110	GU687289
Xanthorhoe decoloraria	BC ZSM Lep 24111	GU687282
Xanthorhoe designata	BC ZSM Lep 02773	HQ601591
Xanthorhoe ferrugata	BC ZSM Lep 24975	GU654942
Xanthorhoe ferrugata	BC ZSM Lep 02817	HQ601592
Xanthorhoe ferrugata	BC ZSM Lep 02816	HQ601593
Xanthorhoe ferrugata	BC ZSM Lep 02815	HQ601594
Xanthorhoe ferrugata	BC ZSM Lep 02804	HQ601595
Xanthorhoe ferrugata	BC ZSM Lep 02778	HQ601596
Xanthorhoe ferrugata	BC ZSM Lep 01262	HQ601597
Xanthorhoe ferrugata	BC ZSM Lep 01247	HQ601598
Xanthorhoe fluctuata	BC ZSM Lep 02803	HQ601599
Xanthorhoe fluctuata	BC ZSM Lep 02801	HQ601600
Xanthorhoe fluctuata	BC ZSM Lep 02736	HQ601601
Xanthorhoe fluctuata	BC ZSM Lep 01239	HQ601602
Xanthorhoe fluctuata	BC ZSM Lep 01238	HQ601603
Xanthorhoe fluctuata	BC ZSM Lep 22425	GU654916
Xanthorhoe fluctuata	BC ZSM Lep 02798	HQ601604
Xanthorhoe incursata	BC ZSM Lep 24115	GU687278
Xanthorhoe montanata	BC ZSM Lep 15097	HQ601605
Xanthorhoe montanata	BC ZSM Lep 15096	HQ601606
Xanthorhoe montanata	BC ZSM Lep 22063	GU686921
Xanthorhoe montanata	BC ZSM Lep 24114	GU687285
Xanthorhoe quadrifasiata	BC ZSM Lep 21383	HQ601607
Xanthorhoe quadrifasiata	BC ZSM Lep 01627	HQ601608
Xanthorhoe quadrifasiata	BC ZSM Lep 01263	HQ601609
Xanthorhoe quadrifasiata	BC ZSM Lep 01242	HQ601610
Xanthorhoe quadrifasiata	BC ZSM Lep 28274	GU707340
Xanthorhoe spadicearia	BC ZSM Lep 01252	HQ601611
Xanthorhoe spadicearia	BC ZSM Lep 01232	HQ601612
Xanthorhoe spadicearia	BC ZSM Lep 01204	HQ601613
Xanthorhoe spadicearia	BC ZSM Lep 24112	GU687283
